# A TMEM16J variant leads to dysregulated cytosolic calcium which may lead to renal disease

**DOI:** 10.1096/fj.202200968R

**Published:** 2022-12-15

**Authors:** Rainer Schreiber, Khaoula Talbi, Jiraporn Ousingsawat, Karl Kunzelmann

**Affiliations:** ^1^ Institut für Physiologie Universität Regensburg Regensburg Germany

**Keywords:** ANO9, anoctamin 9, Ca^2+^ signaling, chronic renal failure, renal transplant, TMEM16J

## Abstract

SIGIRR (single immunoglobulin IL‐1 related receptor), PKP3 (plakophilin 3), and TMEM16J (anoctamin 9), a putative calcium‐activated ion channel and phospholipid scramblase, control the immune response and the extent of inflammation. Variants of SIGIRR/PKP3/TMEM16J lead to severe inflammatory diseases such as pneumonia, enterocolitis, and kidney graft rejection. Meta‐analysis of genome‐wide association studies identified TMEM16J‐T604A as a promotor for chronic kidney disease (CKD), but the disease mechanism and function of TMEM16J remain unknown. Here, we demonstrate TMEM16J as a calcium‐activated calcium‐permeable channel, which is expressed in the endoplasmic reticulum (ER). TMEM16J controls the intracellular distribution of calcium, and inhibits intracellular receptor‐mediated Ca^2+^ signals and Ca^2+^‐dependent activation of ion channels, but augments transcription and release of pro‐inflammatory cytokines. Renal epithelial cells expressing the variant TMEM16J‐T604A show enhanced calcium signals when compared to cells expressing wt‐TMEM16J, and demonstrate spontaneous transcription and release of cytokines. This study identifies TMEM16J as an important regulator of intracellular Ca^2+^ signals, ion channel activity, and cytokine release. TMEM16J may therefore affect immune response in renal tissue and immune cells.

**Abbreviations**
CAAX: cysteine, two aliphatic amino acids plus methionin, serine, alanin, cystein, or glutamin
CKD: chronic kidney disease
ER: endoplasmic reticulum
GWAS: genome‐wide association study
MPTE: mouse primary proximal tubular epithelial
PKP3: plakophilin 3
SIGIRR: single immunoglobulin IL‐1 related receptor
TMEM16J: transmembrane protein 16J

## 1 INTRODUCTION

Exaggerated immune response is inhibited by extra‐ and intracellular mechanisms.^1^ A genetic region encoding single immunoglobulin interleukin‐1 related receptor (SIGIRR), plakophilin 3 (PKP3), and transmembrane 16 protein member J, (TMEM16J, anoctamin 9) was identified as an essential negative regulator of immune response.^2^, ^3^ Common single nucleotide polymorphisms (SNPs) in the SIGIRR‐PKP3‐TMEM16J gene region are associated with tuberculosis, pneumonia, enterocolitis, and kidney graft rejection, and studies of SIGIRR‐deficient mice suggest a central role of SIGIRR for negative regulation of innate immune pathways and adaptive immune response.^3^, ^4^, ^5^, ^6^ Thus, SIGIRR is known to control epithelial homeostasis, inflammation, and tumorigenesis,^6^ while limiting adaptive immunity against kidney grafts.^7^ Importantly, dendritic cells lacking expression of SIGIRR release more IL‐6 upon stimulation with toll‐like receptor 4 (TLR4) ligands or TNF‐α, which supports rejection of renal grafts.^7^

Little is known about the function of TMEM16J, a member of the TMEM16/anoctamin family of ion channels and phospholipid scramblases.^8^ It could form an ion channel, but the evidence was also provided suggesting that it may scramble membrane phospholipids in a Ca^2+^‐dependent manner.^9^, ^10^, ^11^ TMEM16J is expressed in renal proximal tubular epithelial cells and immune‐competent tubulointerstitial cells, such as dendritic cells and T‐cells (https://atlas.kpmp.org/explorer/dataviz). TMEM16J could be, therefore, involved in allograft rejection and renal diseases.^12^ Activation of dendritic cells is strongly supported by vitamin D, while low vitamin D levels in people carrying SIGIRR‐PKP3‐TMEM16J variants are associated with infectious diseases.^13^ Members of the TLR/IL‐1R superfamily mediate ischemia/reperfusion injury and initiate an immune response in transplanted kidneys, a process that is modulated by SIGIRR.^7^ Stanzick and collaborators demonstrated the role of SIGIRR and TMEM16J for chronic renal failure, which is characterized by a reduction in glomerular filtration and increased urinary albumin excretion.^14^, ^15^ A large GWAS meta‐analysis of creatinine‐based estimated glomerular filtration rates (eGFR) in CKD patients was performed by a genetics consortium and the UK Biobank. Prioritization revealed several compelling CKD‐related genes, among them were SIGIRR and TMEM16J.^14^ The TMEM16J variant T604A was one of the top hits related to CKD. Thus compromised function of SIGIRR, PKP3 or TMEM16J may predispose to compromised renal function and an impaired SIGIRR‐PKP3‐TMEM16J cascade may no longer prevent tissue injury due to overshooting initiate immune responses in transplanted kidneys or during postischemic activation of intrarenal myeloid cells.^16^

The immunomodulatory role of SIGIRR at the level of IL‐1 and Toll‐like receptors and its role in inflammatory diseases are well described.^17^, ^18^, ^19^ However, it is unclear whether TMEM16J controls immune response. Our previous work demonstrated the role of TMEM16A in intracellular Ca^2+^ signaling and the release of cytokines.^20^ Here, we examined the function of TMEM16J in renal tubular epithelial cells, and whether TMEM16J controls intracellular Ca^2+^ signals and possibly affects renal immune response. The results could provide a molecular mechanism by which the variant TMEM16J‐T604A may lead to CKD and renal failure.

## 2 MATERIALS AND METHODS

### 2.1 Cell culture and transfection

Human Embryonic Kidney (HEK) 293 cells were cultured in DMEM (Dulbecco's Modified Eagle Medium, Thermo Fisher Scientific, Darmstadt, Germany) with 10% FBS, without antibiotics. immortalized human proximal kidney epithelial (IHKE‐1) cells were cultured in HAM's F12 (Life Technologies/Gibco®, Karlsruhe, Germany) and Dulbecco's modified Eagle's medium (Life Technologies/Gibco®) 1:1, supplemented with 15 mM HEPES (Life Technologies/Gibco®), 100 000 U/L penicillin and 100 mg/L streptomycin (Life Technologies/Gibco®), 10 μg/L epidermal growth factor (Calbiochem, Bad Soden, Germany), 36 μg/L hydrocortisone (Sigma‐Aldrich, Merck KGaA, Darmstadt, Germany), 1% fetal calf serum (Life Technologies/Gibco®), 912 mg/L NaHCO_3_ (Merck), 5 mg/L insulin (Sigma‐Aldrich, Merck), 5 mg/L transferrin (SigmaAldrich, Merck), 5 μg/L Na^+^ selenite (Sigma‐Aldrich, Merck), 55 mg/L Na^+^ pyruvate (Sigma‐Aldrich, Merck) and 2 mM L‐glutamine (Life Technologies/Gibco®). IHKE‐1 cells were transfected with siRNA against TMEM16J (Stealth RNAi, Invitrogen, Thermo Fisher Scientific, Waltham, MA 02451, USA) or scrambled siRNA (Stealth RNAi, Invitrogen, Thermo Fisher Scientific) using standard protocols for Lipofectamine 3000 (Thermo Fisher Scientific). All experiments were performed 48‐72 h after transfection. Successful knockdown of anoctamins was demonstrated by semiquantitative and by real‐time RT‐PCR. Cells were incubated with LPS 100 ng/mL (L9143, Sigma‐Aldrich, Merck) for 24 h. All cells were cultured at 37°C in a humidified atmosphere of 5% (v/v) CO_2_.

### 2.2 Isolation of cortical primary cells

Mice were killed by cervical dislocation after exposure to CO_2_. Kidneys were removed and kept in ice‐cold DMEM/F12 medium (Thermo Fisher Scientific). The renal capsule was removed under germ‐free conditions. The cortex and medulla were separated and chopped into smaller pieces of tissue using a sharp razor blade (Heinz Herenz, Hamburg, Germany). Tissues were incubated in Hanks balanced salt solution/DMEM/F12 (Life Technologies/Gibco®) containing 1 mg/ml collagenase type 2 (Worthington, Lakewood, USA) for 20 min at 37°C. The digested tissue was passed through a 100 μm cell strainer (Merck), transferred to a 50 ml falcon tube, and washed with ice‐cold PBS. After centrifugation at 5100 rpm for 4 min / 4°C, cells were resuspended. After resuspension, the cortical cell pellet was centrifuged at 17500 rpm for 30 min at 4°C through a 45% Percoll (Ge Healthcare GmbH, Munich, Germany) 55% 2X PBS‐Glucose gradient. After washing with ice‐cold PBS, tubular preparations were maintained at 37°C/ 5% CO_2_ in DMEM/F12 supplemented with 1% FBS, 1% Pen/Strep, 1% L‐Glutamine (200 mM), 1% ITS (100x), 50 nM hydrocortisone, 5 nM triiodothyronine, and 5 nM epidermal growth factor (Sigma Taufkirchen, Germany). After 24 h, primary cells grew out from isolated tubules.

### 2.3 Immunohistochemistry, immunofluorescence

Mouse kidneys were fixed by perfusion with 4% paraformaldehyde and post‐fixed in 0.5 mol/L sucrose and 4% paraformaldehyde solution. The paraffin‐embedded tissues were cut at 5 μm on a rotary microtome (Leica Mikrotom RM 2165, Wetzlar, Germany). The sections were de‐waxed and re‐hydrated. For immunohistochemistry, sections were cooked in citrate buffer (pH 6) for 15 min and permeabilized and blocked with 0.04% Triton X‐100 and 5% BSA for 30 min at 37°C. Sections were incubated with primary antibodies against TMEM16J (DPAB23788, Lot: LLE290412, Creative Diagnostics, Shirley, NY 11967, USA), megalin (sc‐515 750, Lot: F2821, Santa Cruz, Dallas, Texas 75 220, USA), calbindin (#300, Lot: 07, Swant, PO Box 327, 1723 Marly 1, Switzerland) and AQP2 (sc‐9882, Lot: D2910, Santa Cruz, Dallas, Texas 75 220, USA) in 0.5% BSA and 0.04% Triton X‐100 overnight at 4°C and subsequent with a secondary donkey anti‐rabbit Alexa 555 (for TMEM16J, ab150074, Lot: GR3241278‐7, Abcam, Berlin, Germany), donkey anti‐goat‐Alexa 488 (for AQP2; A11055, Lot: 1087906), donkey anti‐mouse Alexa 488 (A21202, Lot: 1915874, for calbindin) or donkey anti‐mouse Alexa 660 (A21055, Lot: 1495928, for megalin) IgG (Invitrogen, Thermo Fisher, Scientific, Waltham, MA 02451, USA) for 1 h at 37°C. Sections were counterstained with Hoe33342 (Sigma‐Aldrich, Merck KGaA, Darmstadt, Germany). For analysis of cellular localization, HEK293T cells were transfected with TMEM16J tagged with mcherry or GFP in pcDNA31 or SIGIRR tagged with CFP in pcDNA31. Endoplasmic reticulum (ER) was visualized using ER‐Tracker (BlueWhite DPX, E12353, Lot: 1177457, Invitrogen, Thermo Fisher Scientific). The plasma membrane was stained with FM 4–64 (T13320, Lot: 1702510, molecular probes, Invitrogen, Thermo Fisher Scientific). Immunofluorescence was detected using an Axiovert Observer microscope equipped with ApoTome2 and ZEN 2.6 (blue edition) Software (Carl Zeiss Microscopy Deutschland GmbH, Oberkochen, Germany).

### 2.4 RT‐PCR, real‐time PCR

For semi‐quantitative RT‐PCR or real‐time PCR total RNA from HEK293, IHKE, and primary proximal tubular cells were isolated using NucleoSpin RNA II columns (Macherey‐Nagel, Düren, Germany). Human kidney total RNA was purchased from ThermoFisher Scientific (QS0616, Lot 296633‐000). Total RNA (0.5 μg / 25 μl reaction) was reverse‐transcribed using random primers (Promega, Mannheim, Germany) and M‐MLV reverse transcriptase RNase H Minus (Promega, Mannheim, Germany). Each RT‐PCR reaction contained sense (0.5 μM) and antisense primers (0.5 μM) (Table 1), 0.5 μl cDNA, and GoTaq Polymerase (Promega, Mannheim, Germany). After 2 min at 95°C, cDNA was amplified (targets 30–35 cycles, reference Gapdh 25 cycles) for 30 s at 95°C, 30 s at 56°C and 1 min at 72°C. PCR products were visualized by loading on Midori Green Xtra (Nippon Genetics Europe) containing agarose gels and analyzed using Image J 1.52r (NIH, USA). Bands of interest and Gapdh bands were analyzed by densitometry and the ratio of both bands was taken as a measure for the relative number of transcripts.

**TABLE 1:** RT‐PCR primers used in the present study

Gene accession number	Primer	Size (bp)
human TMEM16A, NM_018043	s: 5′‐ CGACTACGTGTACATTTTCCG as: 5′‐ GATTCCGATGTCTTTGGCTC	445
mouse Tmem16a NM_001242349	s: 5´‐GTGACAAGACCTGCAGCTAC as: 5´‐GCTGCAGCTGTGGAGATTC	406
human TMEM16B NM_001278596	s: 5′‐ GTCTCAAGATGCCAGGTCCC as: 5′‐ CTGCCTCCTGCTTTGATCTC	553
mouse Tmem16b NM_153589	s: 5′‐ CCAGAGGAAAGTCGACTATG as: 5′‐ GGTAGCATTGTCAAAGAAGG	544
human TMEM16C NM_001313726	s: 5´‐CTTCCCTCTTCCAGTCAAC as: 5′‐ AAACATGATATCGGGGCTTG	461
mouse Tmem16c NM_001128103	s: 5′‐ TGATAAAAGAAACACATTTGAAAAGAA as: 5′‐ GAGGCTGATGCTTGTACCAC	611
human TMEM16D NM_001286615	s: 5′‐ GAATGGGACCTGGTGGAC as: 5′‐ GAGTTTGTCCGAGCTTTTCG	713
mouse Tmem16d NM_001277188	s: 5′‐ TGGCTTCATTTTTGCTGTTCT as: 5′‐ GAAGAGCATGCCTGTGTACC	555
human TMEM16E NM_213599	s: 5′‐ GAATGGGACCTGGTGGAC as: 5′‐ GAGTTTGTCCGAGCTTTTCG	713
mouse Tmem16e NM_177694	s: 5′‐ TCCTGAGGAGGCGTCTTATG as: 5′‐ CCCAATCTTTTTCTTCCCCTC	548
human TMEM16F NM_001025356	s: 5′‐ GGAGTTTTGGAAGCGACGC as: 5′‐ GTATTTCTGGATTGGGTCTG	325
mouse Tmem16f NM_001253813	s: 5′‐ CATACGAATCTAACCTTATCTGC as: 5′‐ CATTCTCTGTACAGGAGGTAAC	520
human TMEM16G NM_001370694	s: 5′‐ CTCGGGAGTGACAACCAGG as: 5′‐ CAAAGTGGGCACATCTCGAAG	470
mouse Tmem16g NM_207031	s: 5′‐ TTGGAATCCGAAATGAGGAG as: 5′‐ GTGTGCGGAGGTGAAAGTG	584
human TMEM16H NM_020959	s: 5′‐ GGAGGACCAG CCAATCATC as: 5′‐ TCCATGTCATTGAGCCAG	705
mouse Tmem16h NM_001164679	s: 5′‐ CTTGGAGGACCAGCCAATC as: 5′‐ CTTCTTGTAGCCCTCAGCAC	682
human TMEM16J NM_001012302	s: 5′‐ GCAGCCAGTTGATGAAATC as: 5′‐ GCTGCGTAGGTAGGAGTGC	472
mouse Tmem16j NM_178381	s: 5′‐ CAAGATGTTAAAGGACCAGAAG as: 5′‐ GAAGATATCATTGGCACTACAG	487
human TMEM16K NM_018075	s: 5′‐ GTGAAGAGGAAGGTGCAGG as: 5′‐ GCCACTGCGAAACTGAGAAG	769
mouse Tmem16k NM_133979	s: 5′‐ GGACATGAAGCTTTTGCGCC as: 5′‐ TGGCAAATGCGAGTATGAAC	566
human SIGIRR NM_001135054	s: 5′‐ CTGTCAGAGGTGCTTGTGTC as: 5′‐ GTTCACCAAGAGGTCGGCG	432
mouse Sigirr NM_023059	s: 5′‐ CAGAGATTGTGTCCAGTGTCC as: 5′‐ CTGGGCAGTCGCTATAGGAC	300
human PKP3 NM_007183	s: 5′‐ GTATTCTGAACCCCCTGCTAG as: 5′‐ CGGTGGAGCTTGTTGTACTG	382
mouse Pkp3 NM_019762	s: 5′‐ CTGAACCCCTTGCTGGACC as: 5′‐ CACGGTGGAGCTTGCTGTAC	379
human TLR4 NM_138554	s: 5′‐ CTGCTCTAGAGGGCCTGTG as: 5′‐ CTGGTGTGAGTATGAGAAATG	556
mouse Tlr4 NM_021297	s: 5′‐ GATCTGAGCTTCAACCCCTTG as: 5′‐ CCTCTTAGAGTCAGTTCATGG	527
human IL1R1 NM_000877	s: 5′‐ TGGAGAATGAGCCTAACTTATG as: 5′‐ CTACTTCCATTGTCTCATTAGC	375
mouse Il1r1 NM_008362	s: 5′‐ CTGTGTTAGAGAATGACCCTG as: 5′‐ GTAGACAAGGTCTGAGAACTG	443
human IL6R NM_000565	s: 5′‐ GAGGAAGTTTCAGAACAGTCC as: 5′‐ CGTGGATGACACAGTGATGC	392
mouse Il6ra NM_010559	s: 5′‐ GGACTACCACAGGAAACACAC as: 5′‐ CTTCGTTGTGGCTGGACTTG	402
human IL8R, CXCR1 NM_000634	s: 5′‐ GATCTAAATTTCACTGGCATGC as: 5′‐ GTTTGGATGGTAAGCCTGGC	504
mouse Cxcr2 NM_009909	s: 5′‐ CATGCCACTCAGAGAACCTG as: 5′‐ GCTATGCACACAAACTTGACC	391
human IL6 NM_000600	s: 5′‐ CGGTCCAGTTGCCTTCTCC as: 5′‐ CACTACTCTCAAATCTGTTCTG	383
mouse Il6 NM_031168	s: 5′‐ GAGACTTCCATCCAGTTGCC as: 5′‐ CTGTATCTCTCTGAAGGACTC	420
human IL8, CXCL8 NM_000584	s: 5´‐TGCAGCTCTGTGTGAAGGTG as: 5´‐ACTTCTCCACAACCCTCTGC	227
mouse Cxcl1 NM_008176	s: 5′‐ CGCTCGCTTCTCTGTGCAG as: 5′‐ GCCAGCGTTCACCAGACAG	327
human ATP2B1 NM_001001323	s: 5′‐ CAGGTCCACAGATGCATTACG as: 5′‐ GTTCTTGTTCAATTCGGCTCTG	455
human ATP2B4 NM_001001396	s: 5′‐ CACTATGGAGGTGTACAGAATC as: 5′‐ GTCACTAACACCACGATGATC	359
human, mouse Gapdh NM_001289726	s: 5′‐ GTATTGGGCGCCTGGTCAC as: 5′‐ CTCCTGGAAGATGGTGATGG	200
human ACTB NM_001101	s: 5′‐ CAACGGCTCCGGCATGTG as: 5′‐ CTTGCTCTGGGCCTCGTC	151

Real‐time PCR of cDNA samples was performed in a LightCycler 480 device (Roche, Basel, Switzerland) using specific, intron‐spanning primers (Table 2) and a SYBR® Green mastermix (Takyon, Eurogentec, Belgium). Target gene expression levels were quantified relative to beta‐actin expression under consideration of PCR efficiencies calculated on the basis of standard dilution curves. The specificity of PCR amplifications was verified by agarose electrophoresis and melting curve analysis.

**TABLE 2:** PCR primers used for real‐time PCR

Gene accession number	Primer	Size (bp)
human TMEM16J NM_001012302	s: 5′‐ CAAACCCCAGCTGGAACTC as: 5′‐ GGATCCGGAGGCTCTCTT	61
mouse Tmem16j NM_178381	s: 5′‐ CAGAGCCCCACATTGACC as: 5′‐ CTGGGAACTCTCATCATCCTG	60
human IL6 NM_000600	s: 5′‐ CAAAAGTCCTGATCCAGTTCC as: 5′‐ CAGCAGGCTGGCATTTGTGG	88
mouse Il6 NM_031168	s: 5′‐ CCAGAGATACAAAGAAATGATGG as: 5′‐ GTACTCCAGAAGACCAGAGG	90
human IL8, CXCL8 NM_000584	s: 5′‐ CAGCCTTCCTGATTTCTGCAG as: 5′‐ GTGGAAAGGTTTGGAGTATGTC	104
mouse Cxcl1 NM_008176	s: 5′‐ CAGAAAATTGTCCAAAAGATGC as: 5′‐ GCCAGCGTTCACCAGACAG	93
Human, mouse b‐Actin BC002409.2, NM_007393.5	s: 5′‐ CAACGGCTCCGGCATGTG as: 5′‐ CTTGCTCTGGGCCTCGTC	151

### 2.5 Measurement of [Ca^2+^]_*i*_

Measurement of cytosolic Ca^2+^ changes was performed as described recently.^21^ In brief, HEK293T, IHKE‐1, and cortical primary cells were loaded with 2 μM Fura‐2, AM (BIOZOL, Eching, Germany) in OptiMEM (Gibco, Thermo Fisher Scientific) with 0.02% Pluronic F‐127 (Invitrogen, Thermo Fisher Scientific,) in ringer solution (mmol/l: NaCl 145; KH_2_PO_4_ 0,4; K_2_HPO_4_ 1,6; Glucose 5; MgCl_2_ 1; Ca^2+^‐Gluconat 1,3) for 1 h at room temperature. Fluorescence was detected in cells perfused with Ringer's solution at 37°C using an inverted microscope (Axiovert S100, Zeiss, Germany) and a high‐speed polychromator system (VisiChrome, Puchheim, Germany). Fura‐2 was excited at 340/380 nm, and the emission was recorded between 470 and 550 nm using a CCD camera (CoolSnap HQ, Visitron Systems, Germany). [Ca^2+^]_*i*_ was calculated from the 340/380 nm fluorescence ratio after background subtraction. The formula used to calculate [Ca^2+^]_*i*_ was [Ca^2+^]_*i*_ *= Kd* x (*R*‐*R*_min_)/(*R*_max_‐*R*) x (S_f2_/S_b2_), where *R* is the observed fluorescence ratio. The values *R*_max_ and *R*_min_ (maximum and minimum ratios) and the constant S_f2_/S_b2_ (fluorescence of free and Ca^2+^‐bound Fura‐2 at 380 nm) were calculated using 2 μmol/L ionomycin (Biomol GmbH, Hamburg, Germany) and 5 mmol/L EGTA to equilibrate intracellular and extracellular Ca^2+^ in intact Fura‐2‐loaded cells. The dissociation constant for the Fura‐2•Ca^2+^ complex was taken as 224 nmol/L.^22^ ER Ca^2+^ signals were detected in Ca^2+^ sensor ER‐LAR‐GECO1 (Addgene, Cambridge, MA, USA,^23^) expressing cells. Cells were excited at 560 nm and emission was recorded between 620 ± 30 nm. Control of experiment, imaging acquisition, and data analysis were done with the software package Meta‐Fluor (Universal imaging, USA).

### 2.6 Measurement of IL‐6 and IL‐8 release

IL‐6 and IL‐8 secretion were detected using quantikine colorimetric sandwich ELISA kits (R&D systems, Wiesbaden‐Nordenstadt, Germany). IHKE‐1 cells overexpressing TMEM16J or with silenced TMEM16J were treated with 100 ng/mL lipopolysaccharides (LPS, Sigma). The supernatants were collected after 24 hrs of LPS treatment. Particulates were removed by centrifugation and assay immediately according to the protocol for the company. The signal was detected using the microplate reader NOVOstar (BMG Labtech, Offenburg, Germany).

### 2.7 Patch clamp

Cells were patch clamped when grown on coated glass coverslips. Coverslips were mounted in a perfused bath chamber on the stage of an inverted microscope (IM35, Zeiss) and kept at 37°C. Patch pipettes were filled with a cytosolic‐like solution containing (in mM): KCl 30, K‐Gluconate 95, NaH_2_PO_4_ 1.2, Na_2_HPO_4_ 4.8, EGTA 1, Ca‐Gluconate 0.758, MgCl_2_ 1.03, D‐Glucose 5, ATP 3; pH 7.2. The intracellular (pipette) Ca^2+^ activity was 0.1 μM. The bath was perfused continuously with standard bicarbonate‐free Ringer's solution (in mM: NaCl 145, KH_2_PO_4_ 0.4, K_2_HPO_4_ 1.6, Glucose 5, MgCl_2_ 1, Ca‐Gluconate 1.3) at a rate of 4 ml/min. Patch pipettes had an input resistance of 2–5 MΩ and whole‐cell currents were corrected for serial resistance. Currents were recorded using a patch clamp amplifier EPC9, and PULSE software (HEKA, Lambrecht, Germany) as well as Chart software (AD Instruments, Spechbach, Germany). Cells were stimulated with 1 μM ATP in the absence and presence of TRAM34. In regular intervals, membrane voltage (*V*c) was clamped in steps of 20 mV from −100 to +100 mV from a holding voltage of −100 mV. The current density was calculated by dividing whole‐cell currents by cell capacitance.

### 2.8 Materials and statistical analysis

All compounds used were of the highest available grade of purity and were bought from Sigma‐Aldrich (Merck) unless indicated otherwise. Data are shown as individual traces/representative images and/or as summaries with mean values ± SEM, with the respective number of experiments given in each figure legend. For statistical analysis, paired or unpaired Student's *t*‐test or ANOVA were used as appropriate. A *p*‐value of <.05 was accepted as a statistically significant difference.

## 3 RESULTS

### 3.1 TMEM16J is expressed in the endoplasmic reticulum of mouse proximal tubular epithelial cells

**FIGURE 1:**
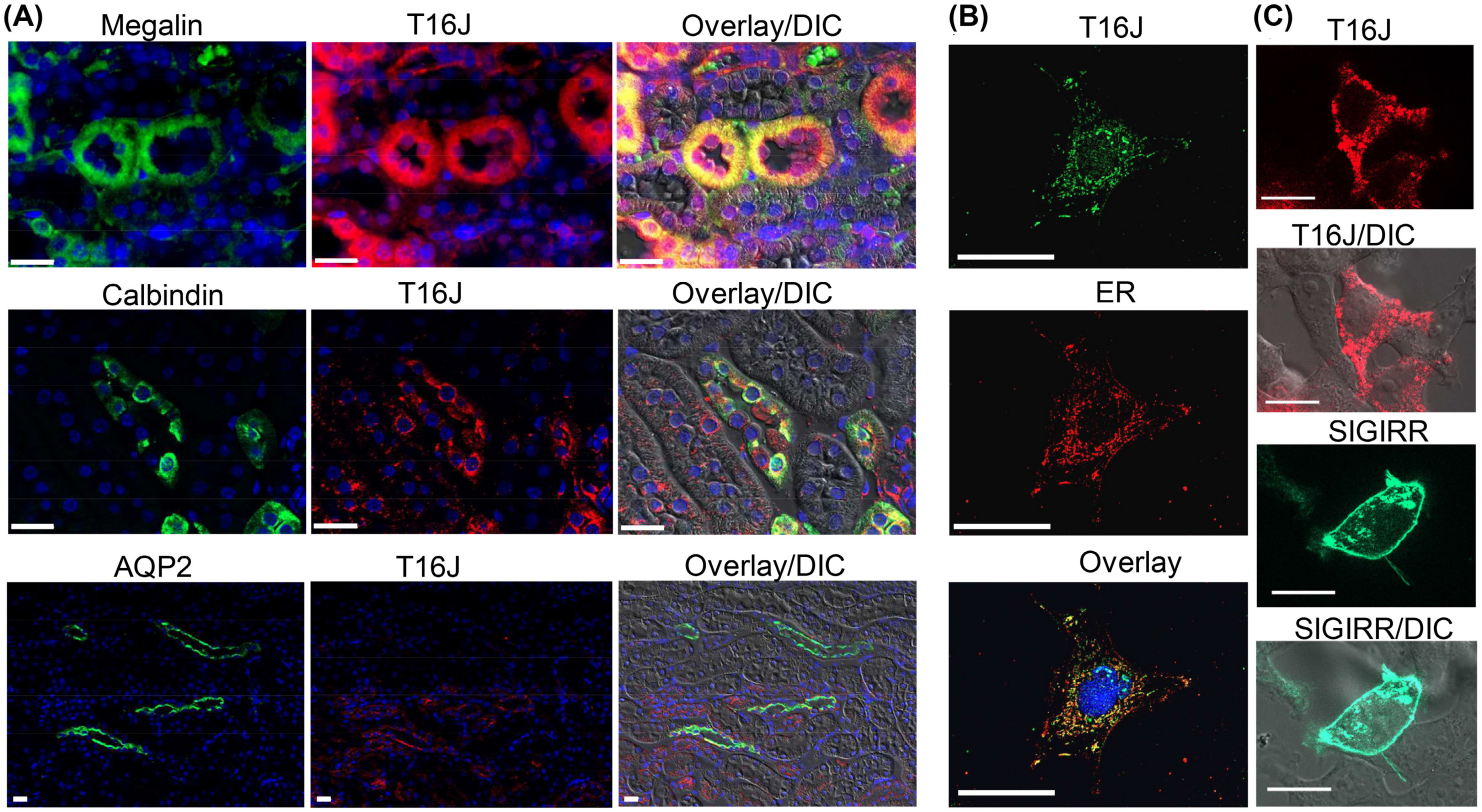
Expression of TMEM16J in the endoplasmic reticulum of renal epithelial cells. (A) Preferential Expression of TMEM16J (red) in proximal tubular epithelial cells of mouse kidney. Co‐staining with markers of proximal tubules (megalin), distal tubule (calbindin), and collecting duct (aquaporin 2; AQP2). TMEM16J was located in intracellular compartments. (B) Co‐staining of TMEM16J (green) with calreticulin (red) identified a preferential location in the endoplasmic reticulum (ER) of mouse primary proximal tubular epithelial cells. (C) Overexpression of TMEM16J and SIGIRR in HEK293 cells indicated expression of TMEM16J in the ER, while SIGIRR is expressed in the plasma membrane.

SIGIRR, TMEM16J, and PKP3 limit immune response and dampen inflammation in various organs including the kidney.^3^, ^4^, ^5^, ^6^, ^24^ The genetically linked immunosuppressive proteins SIGIRR and TMEM16J are strongly expressed in the human kidney (Figure 1, Figure S1). Renal epithelial SIGGIR expression and function have been demonstrated previously, however, the role of TMEM16J in the kidney is unknown. Immunocytochemistry of TMEM16J in mouse kidneys identified expression in the cortex and medulla as indicated by co‐staining with the marker proteins megalin (proximal tubule), calbindin (distal tubule), and aquaporin 2 (AQP2; collecting duct). The most prominent expression was found in the proximal tubule (Figure 1A). In isolated mouse primary proximal tubular epithelial (MPTE) cells, TMEM16J was found to be expressed in the endoplasmic reticulum (ER), but not in the plasma membrane (Figure 1B). Similarly, overexpressed TMEM16J in HEK293 cells was also localized in the ER, while SIGIRR was expressed in the plasma membrane (Figure 1C). We tried different antibodies to quantify the expression of endogenous TMEM16J. While clean Western blot signals were obtained for TMEM16J overexpressed in HEK293 cells, all antibodies behaved non‐specific when used for the detection of endogenous TMEM16J in IHKE‐1 cells by Western blotting (Figure S9).

### 3.2 TMEM16J may operate as an ER‐localized Ca^2+^‐leak channel

**FIGURE 2:**
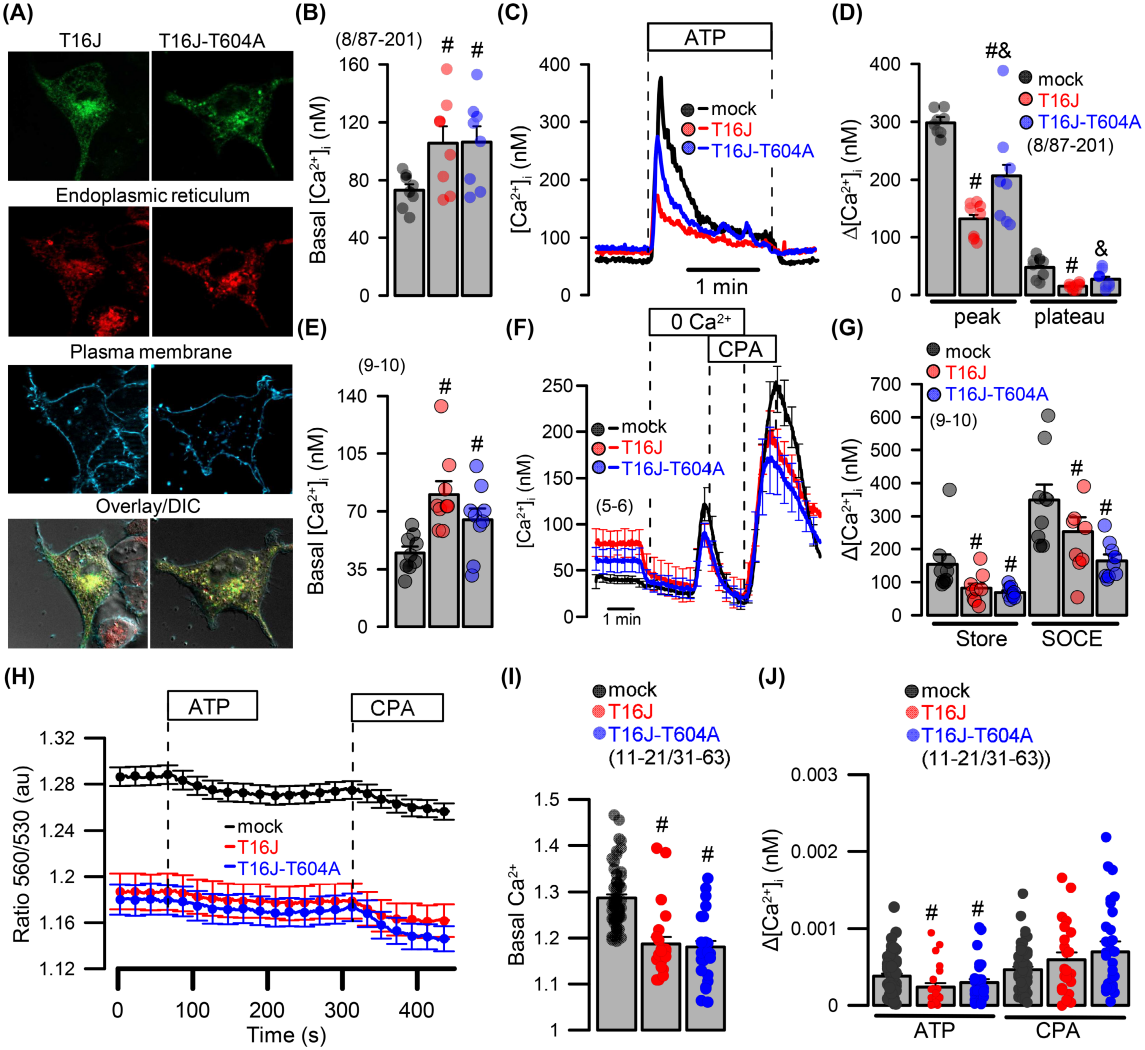
TMEM16J and the variant TMEM16J‐T604A attenuate intracellular Ca^2+^ signals by lowering ER‐Ca^2+^ load. (A) Life co‐staining of the ER (red, ER‐tracker) and the plasma membrane marker (cyan FM4‐64; Molecular Probes) identified expression (green, GFP) of TMEM16J (left) and TMEM16J‐T604A (right) predominantly in the ER, but not in the plasma membrane of overexpressing HEK293 cells. (B) Fura2 Ca^2+^‐measurements indicated higher basal intracellular Ca^2+^ levels in HEK293 cells expressing TMEM16J or TMEM16J‐T604A, when compared to mock‐transfected cells. (C and D) ATP (100 μM) induced ER Ca^2+^ store release was attenuated by TMEM16J and to a lesser extent by TMEM16J‐T604A. (E–G) Basal Ca^2+^ levels were inhibited significantly by removal of extracellular Ca^2+^ (0 Ca^2+^) in TMEM16J and TMEM16J‐T604A‐expressing cells, but not in mock‐transfected cells. ER store release induced by the SERCA‐inhibitor CPA (10 μM) and store‐operated Ca^2+^ influx (SOCE) upon re‐addition of extracellular Ca^2+^ were attenuated in TMEM16J and TMEM16J‐T604A‐expressing cells. (H–J) ER‐Ca^2+^ measured with the dye ER‐LAR‐GECO1, indicated lower ER‐Ca^2+^ levels and attenuated ATP‐induced Ca^2+^ release for both TMEM16J and TMEM16J‐T604A‐expressing cells. Mean ± SEM (number of coverslips analyzed/number of individual experiments). ^#^Significant difference when compared to mock (*p* < .05; ANOVA).

Similar to renal epithelial cells, TMEM16J overexpressed in HEK293 cells is also located in the ER, but not in the plasma membrane (Figure 2A).^9^ ER‐location was identical for both wild type TMEM16J (T16J) and for the TMEM16J variant TMEM16J‐T604A (T16J‐T604A), which is associated with chronic kidney disease.^14^ We previously described the modulation of receptor‐mediated Ca^2+^ signaling by TMEM16 proteins^20^ and therefore compared the effects of T16J and T16J‐T604A on intracellular Ca^2+^. Basal cytosolic Ca^2+^ levels (basal [Ca^2+^]_*i*_) were slightly enhanced by both T16J and T16J‐T604A (Figure 2B). In contrast, ATP‐stimulated peak Ca^2+^ and store‐operated Ca^2+^ influx (SOCE) were reduced in cells expressing T16J. The effects on basal [Ca^2+^]_*i*_, peak Ca^2+^, and SOCE were attenuated in cells expressing T16J‐T604A (Figure 2C,D). When emptying the ER Ca^2+^ stored by the SERCA‐pump inhibitor cyclopiazonic acid (CPA), both Ca^2+^ release and SOCE were reduced in T16J‐T604A‐expressing cells (Figure 2E–G).

We measured directly intra‐ER Ca^2+^ concentrations ([Ca^2+^]_ER_) using the Ca^2+^ sensor ER‐LAR‐GECO1 and found a clearly reduced basal [Ca^2+^]_ER_ in both T16J and T16J‐T604A‐expressing cells. Moreover, ATP‐induced Ca^2+^ release was attenuated for both T16J and T16J‐T604A, while subsequent CPA‐induced release was not different for mock‐transfected cells (Figure 2H–J). The data suggest that T16J operates as an ER Ca^2+^ leak channel that lowers [Ca^2+^]_ER_.

### 3.3 Enhanced activity of PMCA in the presence of TMEM16J curtails intracellular Ca^2+^ signals

**FIGURE 3:**
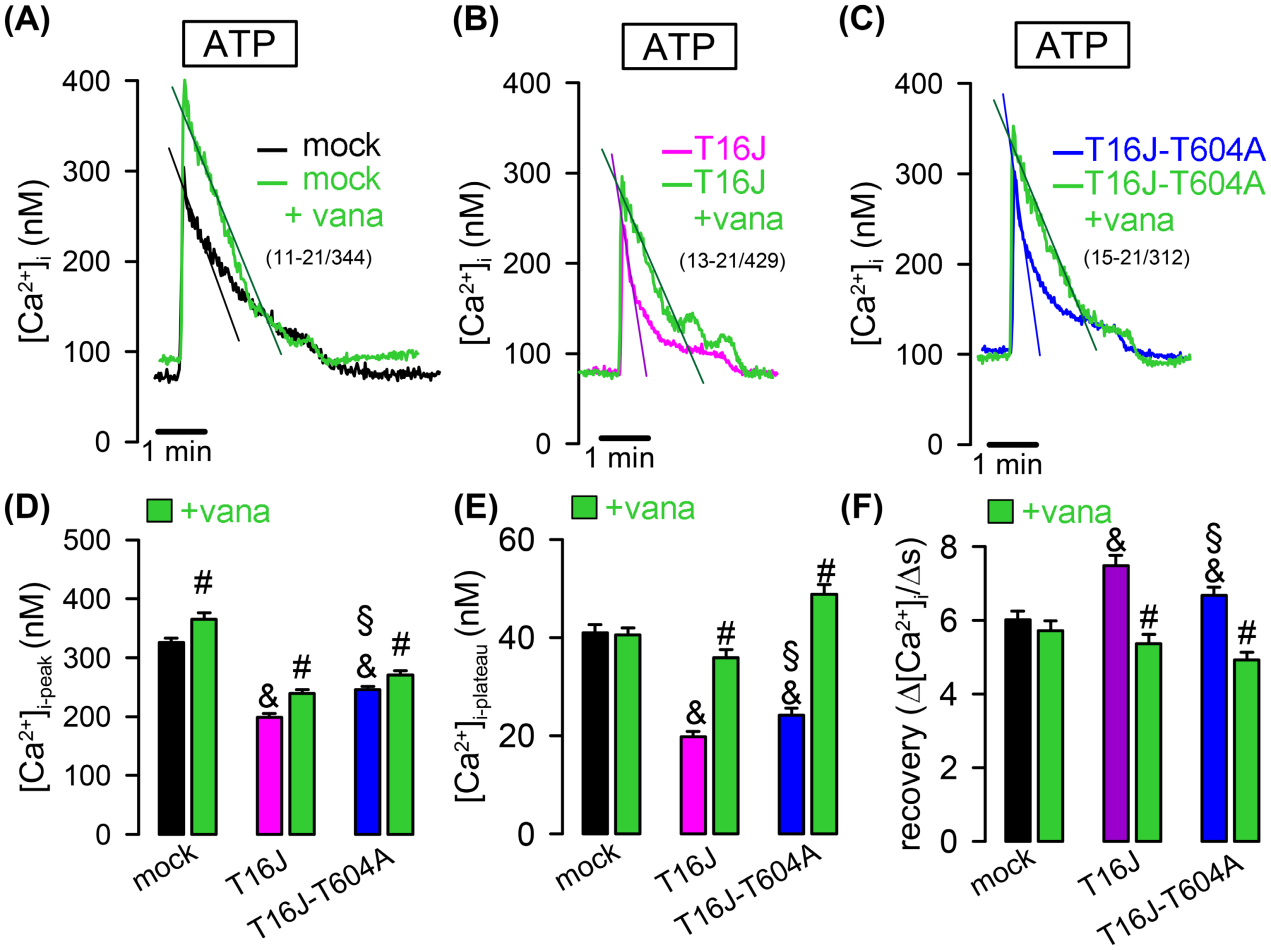
Enhanced PMCA‐activity in cells expressing TMEM16J and TMEM16J‐T604A. (A–C) Original recordings of the intracellular Ca^2+^ concentrations measured by Fura2 in mock‐transfected and TMEM16J or TMEM16J‐T604A expressing HEK293 cells in the absence or presence of the PMCA‐inhibitor vanadate (vana; 200 μM). (D–F) Vanadate enhanced the ATP‐induced plateau and prolonged the recovery from ATP‐induced Ca^2+^ increase in TMEM16J or TMEM16J‐T604A‐expressing cells but not in mock‐transfected cells. Because of the large number of individual observations, the scatter blot has been omitted. Mean ± SEM (number of coverslips analyzed/number of individual experiments). ^#^Significant difference when compared to mock (*p* < .05; ANOVA).

T16J interacts with the scaffold protein DLG1, which controls the activity of the plasma membrane Ca^2+^‐ATPase (PMCA) (http://www.interactome‐atlas.org). PMCA is ubiquitously expressed and plays a key role in fine‐tuning the magnitude and duration of intracellular Ca^2+^ signals following the activation of G‐protein‐coupled receptors (GPCRs).^25^ PMCA is also expressed in HEK293, IHKE‐1and MPTE cells (Figure S2). A signal compartment close to the plasma membrane containing T16J, DLG1, and PMCA could lead to the activation of PMCA and curtail Ca^2+^ signals as observed in T16J‐expressing cells. In fact, HEK293 cells expressing T16J or T16J‐T604 showed short‐lived Ca^2+^ signals upon stimulation of GPCRs by reducing the duration of the Ca^2+^ plateau (Figure 3A–C). We found that vanadate, an inhibitor of PMCA, enhanced plateau‐Ca^2+^ and prolonged Ca^2+^ signals to Ca^2+^ plateau durations found in the absence of T16J (Figure 3D–F). It should be mentioned that experiments with the more specific PMCA‐inhibitor caloxin 1B1 also prolonged Ca^2+^ signals in cells expressing TMEM16J (data not shown). Taken together, the data suggest upregulation of PMCA function in the presence of TMEM16J, possibly by relocating local intracellular [Ca^2+^]_*i*_, and activating calmodulin, which disinhibits PMCA.^25^

### 3.4 Ca^2+^‐dependent activation of TMEM16J which is compromised in the variant TMEM16J‐T604A

**FIGURE 4:**
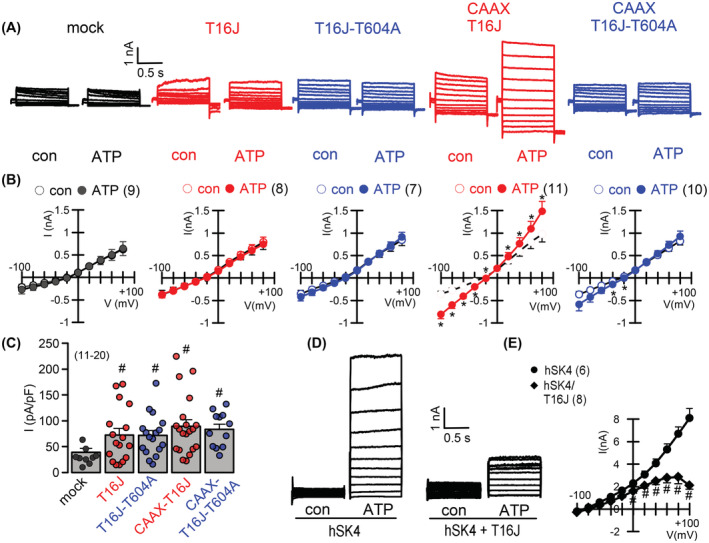
TMEM16J is forming a Ca^2+^ permeable ion channel in the endoplasmic reticulum that is impaired in the variant TMEM16J‐T604A. (A and B) Whole‐cell current overlays of ion currents and corresponding current/voltage relationships before and after stimulation with ATP (100 mM). TMEM16J and TMEM16J‐T604A without or with C‐terminal CAAX motif (KKKKSKTKCVIM) were expressed in HEK293 cells and compared with mock‐transfected cells. Whole‐cell currents could be activated by ATP only in TMEM16J‐T604A‐expressing cells. (C) Basal current densities (at clamp voltages of +100 mV) indicated enhanced basal currents in TMEM16J, CAAX‐TMEM16J, and TMEM16J‐T604A, CAAX‐TMEM16J‐T604A‐expressing cells. (D and E) Whole cells currents and corresponding I/V curves in cells expressing hSK4 or coexpressing hSK4 and TMEM16J. Ca^2+^‐dependent activation of hSK4 by purinergic stimulation (100 μM ATP) was strongly attenuated by coexpression of TMEM16J. Mean ± SEM (number of cells). *Significant activation ATP (paired *t*‐test). ^#^Significantly different from mock and hSK4 (*p* < .05; ANOVA and unpaired *t*‐test, respectively).

Endogenous TMEM16J in MPTE cells and overexpressed TMEM16J in HEK293 cells are located in the ER. In fact, using patch‐clamp recordings we did not measure additional whole‐cell membrane currents in HEK293 cells overexpressing T16J or T16J‐T604A (Figure 4). However, targeting of intracellular transmembrane proteins to the plasma membrane can be induced by C‐terminal fusion to a CAAX (cysteine, two aliphatic amino acids plus methionin, serine, alanin, cystein, or glutamin) motif.^26^ However, despite fusion to CAAX, a substantial portion of overexpressed CAAX‐T16J remained intracellular. Nevertheless, some CAAX‐T16J escaped to the plasma membrane.^26^ We detected membrane expression of CAAX‐T16J using combined GFP‐fluorescence and patch clamp (CAAX‐T16J‐CFP) and found activation of whole‐cell currents by an increase in intracellular Ca^2+^ upon stimulation with the purinergic agonist ATP or with the Ca^2+^ ionophore ionomycin^26^ (Figure 4A,B). In contrast, ATP did not activate the variant CAAX‐T16J‐T604A (CAAX‐T16J‐T604A‐CFP), indicating a defect in Ca^2+^‐dependent activation of T16J‐T604A. However, a closer inspection of the calculated current densities revealed enhanced constitutive (basal) currents in cells expressing T16J or T16J‐T604A, independent of CAAX (Figure 4C). Higher basal currents correspond to higher basal Ca^2+^ levels found in cells expressing T16J or T16J‐T604A (Figure 2B,E). The results suggest that both T16J or T16J‐T604A produce an ER‐Ca^2+^ leak that activates TMEM16F (T16F) expressed endogenously in the plasma membrane of HEK293 cells.^27^ Activation of endogenous T16F by the ER‐localized T16J paralog T16D (TMEM16D, ANO4) has been shown previously.^20^ T16D was also described as a bona fide Ca^2+^‐dependent non‐selective cation channel.^28^ In summary, the data suggest that both T16J and the variant T16J‐T604A form ER‐localized Ca^2+^ permeable ion channels providing a basal ER Ca^2+^ leak. While T16J is further activated by an increase in cytosolic Ca^2+^, T16J‐T604 may have lost its Ca^2+^ regulation, or, alternatively, the Ca^2+^ response curve was shifted to higher cytosolic Ca^2+^ concentrations.

We further examined the effect of attenuated ER Ca^2+^‐store filling in the presence of T16J. The Ca^2+^‐activated K^+^ channel KCa3.1 (KCNN4; SK4) is activated by stimulation of GPCRs and IP3‐mediated Ca^2+^ store release. KCNN4 plays a pronounced role in innate and adaptive immunity.^29^, ^30^ We examined the activation of overexpressed hSK4 channels by purinergic stimulation with ATP (Figure 4D,E). Large hSK4 whole‐cell currents were activated by ATP, which strongly hyperpolarized the membrane voltage. In contrast, when coexpressed with T16J, activation of hSK4 was strongly attenuated due to T16J‐mediated reduced ER‐store filling, and consecutive reduced IP3‐mediated Ca^2+^ store release. Thus, T16J attenuates receptor‐mediated Ca^2+^ signaling and inhibits activation of Ca^2+^‐regulated K^+^ channels that are essential for the proper activation of immune cells. Similarly, activation of TMEM16A Cl^−^ channels were compromised in the presence of T16J (data not shown).

### 3.5 Renal epithelial cells expressing TMEM16J‐T604A present compromised Ca^2+^ signaling leading to constitutive transcription and release of cytokines

**FIGURE 5:**
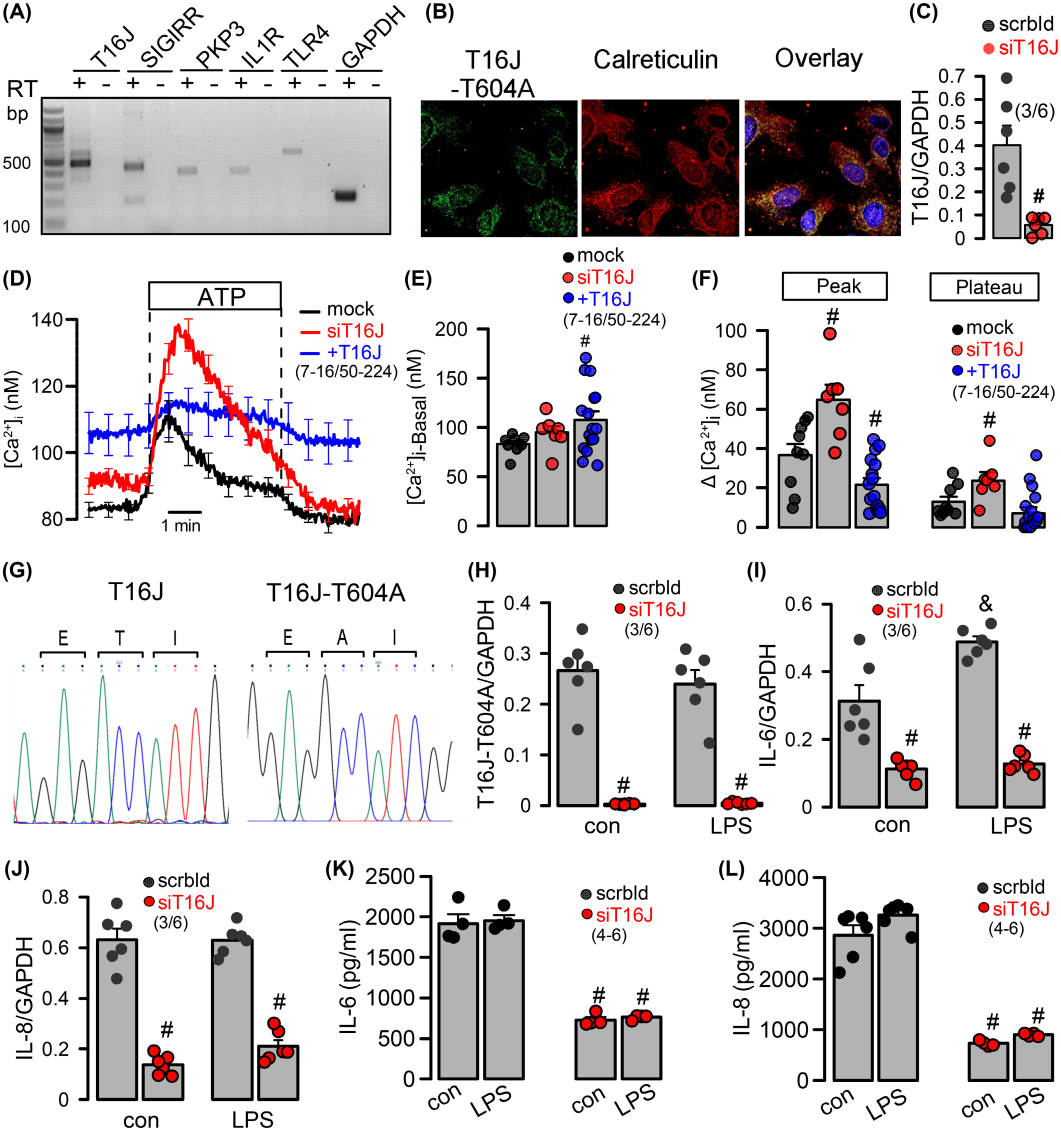
Renal epithelial cells expressing T604A‐TMEM16J present attenuated Ca^2+^ signaling with constitutive transcription and release of cytokines. (A) RT‐PCR indicating expression of TMEM16J, SIGIRR and PKP3 as well as IL‐1R and TLR4 in IHKE1 renal proximal‐tubular epithelial cells. (B) Immunocytochemistry indicated expression of endogenous TMEM16J‐T604A in the ER (stained by calreticulin) of IHKE1 cells. (C) Semiquantitative RT‐PCR indicating suppression of TMEM16J‐T604A expression by siRNA. (D) ATP (100 μM) induced an increase of intracellular Ca^2+^ in mock‐transfected IHKE1 cells (black), and cells treated with siRNA‐TMEM16J (red) or overexpressing TMEM16J (blue). (E) Basal Ca^2+^ levels in IHKE1 cells transfected with siRNA‐TMEM16J or TMEM16J. (F) ATP‐induced store release (peak) and SOCE (plateau) in siRNA‐TMEM16J or TMEM16J transfected cells. (G) Sequencing identifying homozygous expression of TMEM16J‐T604A in IHKE1 cells. (H) Semiquantitative RT‐PCR indicating suppression of TMEM16J‐T604A expression by siRNA in IHKE1 cells, under control conditions and in the presence of LPS. (I and J) Expression of endogenous IL‐6 and IL‐8 in the presence or absence (si‐T16J) of TMEM16J‐T604A. (K and L) Release of IL‐6 and IL‐8 by IHKE1 cells in the presence or absence (siT16J) of TMEM16J‐T604A. Mean ± SEM (number of coverslips/cells or number of assays). *significant activation ATP (paired *t*‐test). ^#^Significant difference to scrambled (scrbld) or mock (*p* < .05; ANOVA and unpaired *t*‐test, respectively).

We examined whether regulation of intracellular Ca^2+^ signals by endogenous TMEM16J is present in IHKE‐1 renal epithelial cells. IHKE‐1 cells express T16J and SIGIRR along with Toll‐like receptor 4 (TLR4) (Figure 5A, Figure S3). Similar to MPTE cells, T16J is also expressed in the ER membrane of IHKE‐1 cells (Figure 5B). Expression of T16J was potently downregulated by siRNA (Figure 5C, Figure S10). When stimulated with ATP, an increase in intracellular Ca^2+^ was clearly detectable and was even enhanced after the knockdown of T16J (Figure 5D–F). In contrast, additional overexpression of T16J augmented basal Ca^2+^ concentrations but strongly attenuated ATP‐induced Ca^2+^ increase. We sequenced T16J and found that IHKE‐1 cells were homozygous for T16J‐T604A (Figure 5G). This was somewhat surprising as the A‐variant at position 604 of T16J (T16J‐T604A) has a frequency of only 3.6%.^14^ Even more surprising, the sequencing of two other proximal tubular epithelial cell lines, HK‐2 and SA7K, also showed homozygosity for T604A (not shown). We speculate that T604A might provide a selection or growth advantage. However, we did not detect a change in cell proliferation or cell survival upon the knockdown of T16J–T604A (Figure S4).

Because the expression of TMEM16J correlates inversely to immune response and inflammation,^2^, ^3^ we examined whether transcription and/or release of interleukins is affected by the expression of T16J‐T604A in IHKE‐1 cells. Semiquantitative (SQ) mRNA‐analysis and real‐time PCR of IL‐6 and IL‐8 surprisingly revealed constitutive high levels for both pro‐inflammatory cytokines, which was only slightly further enhanced by stimulation of TLR‐4 receptors with lipopolysaccharides (LPS) (Figure 5iI,J, Figure 8). Remarkably, the knockdown of T16A‐T604A strongly attenuated the expression of IL‐6 and IL‐8 (Figure 5H–J). All results from SQ RT‐PCR were confirmed by real‐time RT‐PCR (Figure S8). Corresponding to high constitutive transcription of IL‐6 and IL‐8, IHKE‐1 cells demonstrated high basal release of IL‐6 and IL‐8, which, again, was not further enhanced by LPS (Figure 5K,L). As expected, siRNA‐knockdown of T16J‐T604A strongly inhibited the release of both cytokines. Thus, the presence of T16J‐T604A induces transcription and release of pro‐inflammatory cytokines in IHKE‐1 cells.

### 3.6 Primary renal epithelial cells expressing TMEM16J show no constitutive release of cytokines and LPS‐induced release is not inhibited by knockdown of TMEM16J

**FIGURE 6:**
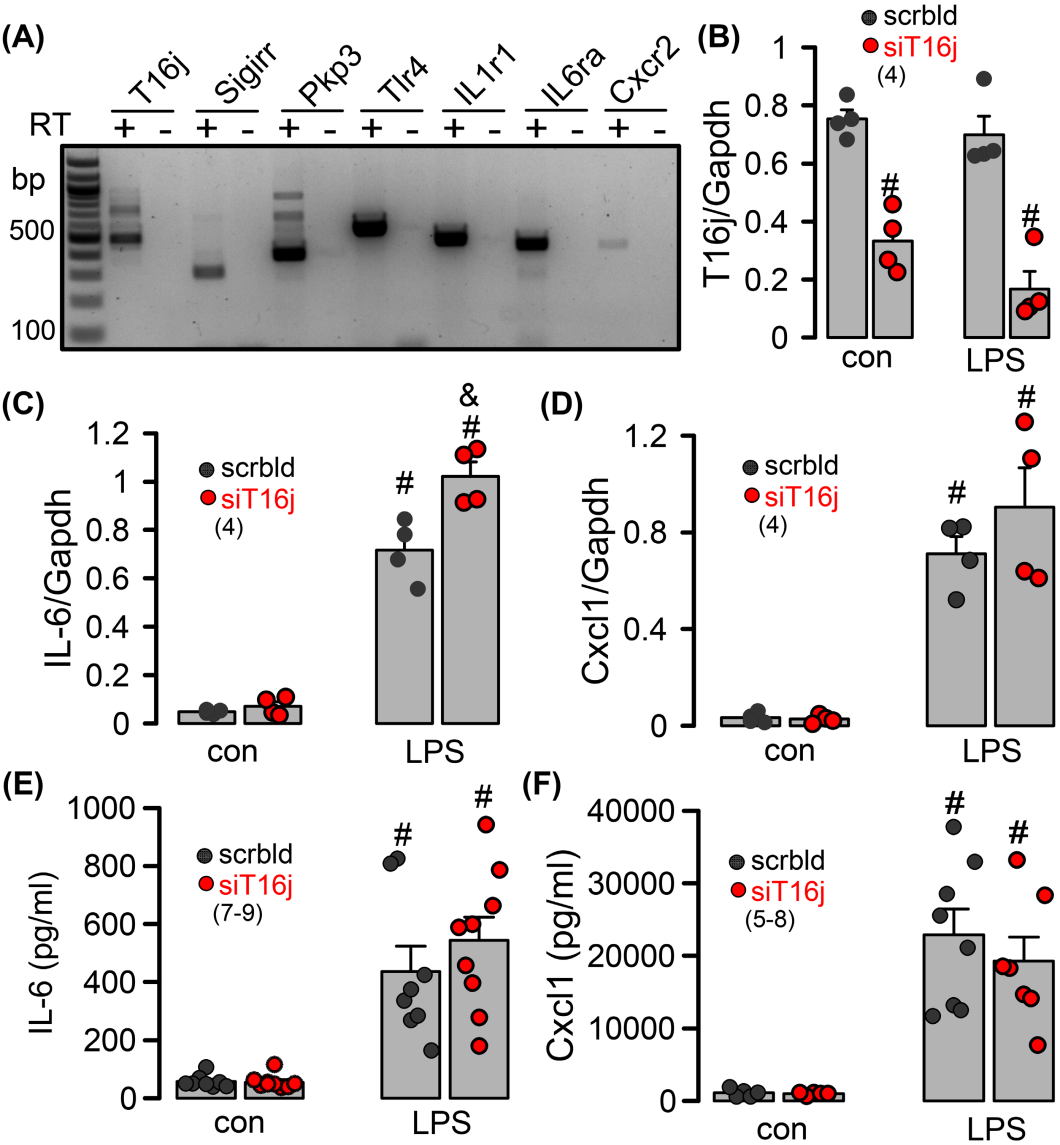
LPS is required for interleukin release in primary renal epithelial cells which is not inhibited by the knockdown of TMEM16J. (A) RT‐PCR indicating expression of TMEM16J, SIGIRR, and PKP3, and cytokine receptors/TLR4 in mouse primary renal tubular epithelial (MPTE) cells. + and – refer to the presence or absence of reverse transcriptase. (B) Semiquantitative RT‐PCR indicating suppression of TMEM16J‐expression by siRNA. (C and D) Expression of endogenous IL‐6 and Cxcl1 in MTPE cells in the presence or absence of TMEM16J, and before and after stimulation with LPS. (E and F) Release of IL‐6 and Cxcl1 by MPTE cells in the presence or absence of TMEM16J, and before and after stimulation with LPS. Mean ± SEM (number of assays). ^#^Significant difference compared to control (*p* < .05; unpaired *t*‐test). ^&^Significant difference compared to scrbld (*p* < .05; unpaired *t*‐test).

**FIGURE 7:**
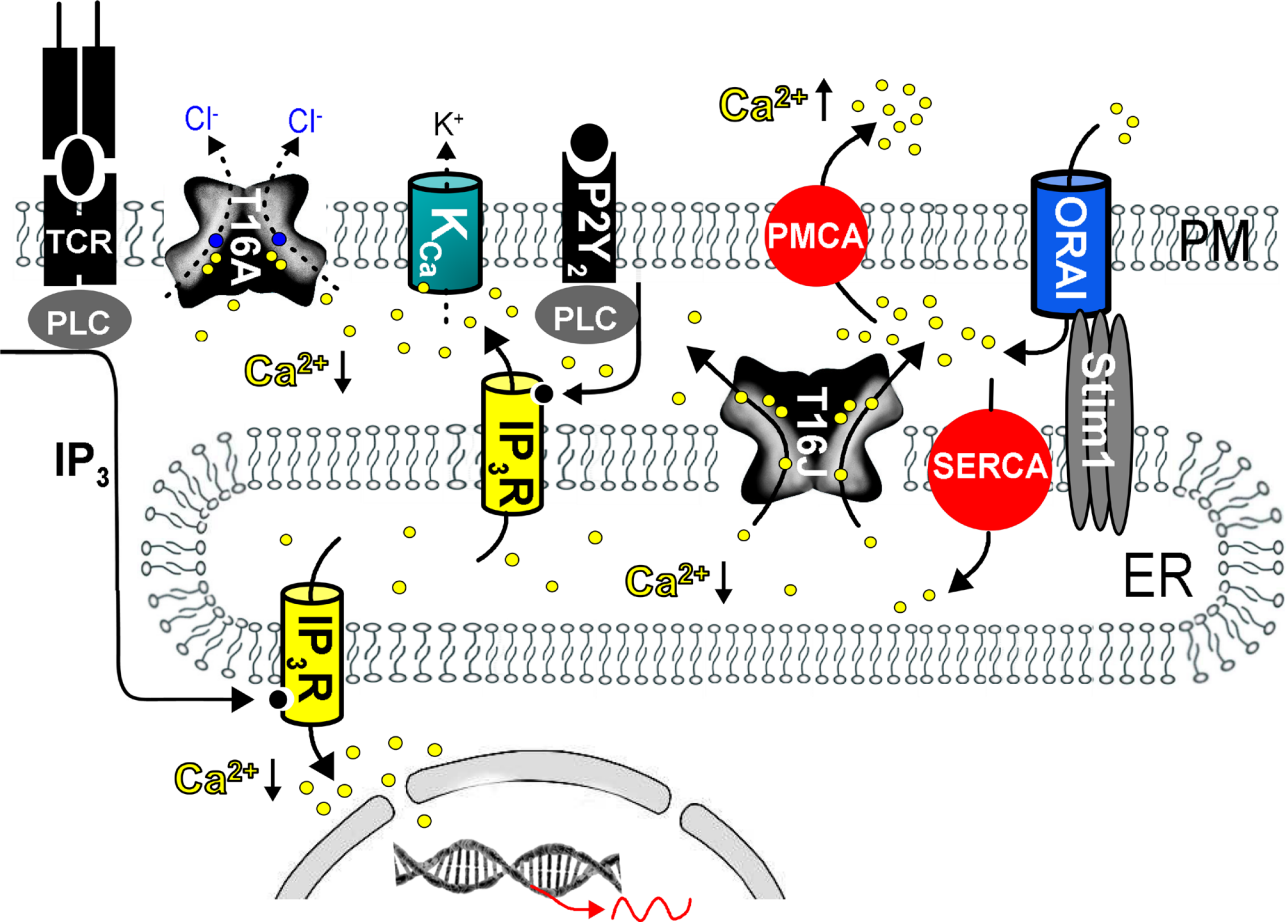
Model for attenuated Ca^2+^ signaling and suppression of immune response by TMEM16J. TMEM16J (T16J) may operate as a Ca^2+^‐activated Ca^2+^ leak channel in the endoplasmic reticulum (ER), which reduces the loading of the ER Ca^2+^ store. Ca^2+^ transport out of the cell by the plasma membrane Ca^2+^‐ATPase (PMCA) is enhanced, thereby contributing to the attenuation of Orai/Stim1/SERCA‐mediated refill of the ER store. Lower store filling leads to attenuated GPCR/IP_3_ induced Ca^2+^ release, and consequently to reduced Ca^2+^‐dependent activation of ion channels like SK4 (K_Ca_) and TMEM16A (T16A), and limited Ca^2+^‐mediated transcription of inflammatory mediators. In T‐cells and dendritic cells, T‐cell receptor (TCR) and P2Y2‐mediated Ca^2+^ signaling, respectively, may be attenuated, leading to lower cytokine production and limited immune response and inflammation.^46^, ^47^, ^48^

Constitutive high release of IL‐6 and IL‐8 by T16J‐T604A expressing IHKE‐1 cells asks for comparison with proximal renal epithelial cells expressing (wild type) T16J. As mentioned, all proximal tubular cell lines (IHKE‐1, HK‐2, and SA7K) express the variant T604A. We, therefore, examined mouse primary proximal tubular epithelial (MPTE) cells, which express mouse wild type T16J (mouse T595 corresponds to human T604). Notably, MPTE cells express the proteins of the Tmem16j‐Sigirr‐Pkp3 gene region along with Tlr4 (Figure 6A). T16j could be successfully downregulated by siRNA in both control MPTE cells and LPS‐activated MPTE cells, as shown by SQ and real‐time RT‐PCR (Figure 6B, Figure 8). In sharp contrast to T16J‐T604A expressing IHKE‐1 cells, MPTE cells did not show a constitutive transcription of IL‐6 or Cxcl1 (corresponding to human IL‐8), and cytokine release was negligible under control conditions (Figure 6C–F). LPS markedly increased transcription and cellular release of IL‐6 and CXCL1, independent of expression of T16j. All results from SQ RT‐PCR were confirmed by real‐time RT‐PCR (Figure S8). Taken together, the present results demonstrate the role of TMEM16J for ER‐Ca^2+^ store‐filling and intracellular Ca^2+^ signaling, which affects gene transcription and release of pro‐inflammatory cytokines (Figure 7). Abnormal intracellular Ca^2+^ signaling in people carrying the T16J‐T604A variant may lead to chronic renal disease.^14^

## 4 DISCUSSION

### 4.1 TMEM16J is an ER‐localized Ca^2+^‐permeable ion channel that has reduced activity when expressed as a T604A variant

SIGIRR is highly expressed in epithelial cells from the kidney, gut, and liver, and is also present in immune cells such as dendritic cells and T‐cells.^1^ TMEM16J, a putative ion channel or phospholipid scramblase,^8^ is located in the same genetic region as SIGIRR. Common polymorphisms in the PKP3‐SIGIRR‐TMEM16J gene region are associated with susceptibility to diverse inflammatory diseases like tuberculosis, pneumonia, enterocolitis, psoriasis, chronic renal disease, and many other inflammatory conditions.^3^, ^5^, ^14^, ^31^ This suggests that similar to SIGIRR, also TMEM16J acts as a negative regulator of inflammation and immunity.^32^ The present study presents a possible molecular mechanism by which TMEM16J dampens tissue inflammation elicited by the activation of Toll‐like or interleukin‐1 receptors. A previous study proposed TMEM16J as a cAMP‐activated Ca^2+^ permeable nonselective ion channel.^9^ Here, we show that TMEM16J is an intracellular, ER‐localized ion channel with negligible expression in the plasma membrane. It was possible to push the plasma membrane expression of TMEM16J, by attaching a CAAX motif.^26^ CAAX‐TMEM16J was activated by GPCR‐mediated Ca^2+^ increase, while CAAX‐TMEM16J‐T604A could not be activated. Homology modeling of TMEM16J suggests a location of T604 in the transmembrane domain (TMD) 9, near the putative Ca^2+^ binding site, which may possibly affect Ca^2+^ binding and opening of the channel (Figure S5). It has been demonstrated that binding of the compound canthaxanthin to TMD9 activates the TMEM16J paralogue TMEM16A, probably by changing its Ca^2+^ sensitivity.^33^ We, therefore, speculate that TMD9 in TMEM16J may similarly affect the Ca^2+^ sensitivity of TMEM16J. In contrast, we did not detect activation by an increase in intracellular cAMP using 3‐Isobutyl‐1‐methylxanthine (IBMX; 100 μM) and forskolin (2 μM) (Figure S6).

### 4.2 TMEM16J controls intracellular Ca^2+^ signaling

The data highlight the effect of TMEM16J on intracellular Ca^2+^ signals. Ca^2+^ loading of the ER Ca^2+^ store is lowered in the presence of TMEM16J. While TMEM16J can be further activated by an increase in intracellular Ca^2+^, this was not observed for TMEM16J‐T604A (Figure 4). Direct measurement of [Ca^2+^]_ER_ using the Ca^2+^ sensor ER‐LAR‐GECO1 exhibited lower ER‐Ca^2+^ for both TMEM16J and TMEM16J‐T604A. ER‐LAR‐GECO1 measurements could not resolve differences between cells expressing TMEM16J and TMEM16J‐T604A, probably due to the low Ca^2+^ sensitivity of ER‐LAR‐GECO1 for Ca^2+^.^20^ According to the present data TMEM16J (and possibly also TMEM16D and TMEM16H) may operate as Ca^2+^‐activated ER Ca^2+^‐leak channels.^28^, ^34^, ^35^ Lower ER Ca^2+^ load with TMEM16J and TMEM16J‐T604A results in enhanced Ca^2+^ influx under control (non‐stimulated) conditions, as shown by the removal of extracellular Ca^2+^ (Figure 2F,G). Unexpectedly, store‐operated Ca^2+^ entry after ER store emptying by CPA was also attenuated in the presence of TMEM16J or TMEM16J‐T604A. Notably, Jha et al demonstrated that the TMEM16J‐paralogue TMEM16H tethers the ER to a phosphatidylinositol‐4,5‐bisphosphate (PI_(4,5)_P_2_) rich compartment that contains Orai/Stim1 and the Ca^2+^‐pump PMCA.^34^ This compartment allows tight communication of Ca^2+^ transporting proteins to control the intensity of Ca^2+^ signaling. Because TMEM16H, TMEM16J, and TMEM16D similarly attenuate receptor‐mediated Ca^2+^ signals,^20^ we could speculate that TMEM16J and TMEM16D have a similar function as TMEM16H. Moreover, homologous proteins such as yeast TMEM16A‐paralogue Ist2 and the Ca^2+^‐activated Cl^−^‐channel TMEM16A also operate as ER‐PM tethers.^36^ TMEM16J (and possibly TMEM16D) may therefore operate as ER‐PM tethers, although a search for potential PIP_2_/membrane interaction sides did not reveal a cluster of positively charged amino acids in either C‐ or N‐ terminus of TMEM16J (Figure S7).^37^ Compartmentalized organization of Ca^2+^ transporting proteins by TMEM16J may shorten Ca^2+^ signals due to the activation of PMCA. Thus, in the presence of TMEM16J, the ER may translocate or may be tethered to an ER‐PM junction that contains PMCA, similar to TMEM16H (Figure 7). Such a scenario may be further supported by the interaction of TMEM16J with the PDZ‐domain protein discs large homolog 1 (Dlg1), a protein that is required for immune cell polarity and regulation of PMCA.^25^, ^38^, ^39^, ^40^, ^41^ As suggested for TMEM16H,^34^ absence or compromised function of TMEM16J and failure to build ER/PM compartments may result in runaway Ca^2+^ responses and renal pathology.

### 4.3 Loss of function mutations in TMEM16J cause exaggerated Ca^2+^ signals and organ disease

The present data show that TMEM16J dampens receptor‐mediated Ca^2+^ signals and that attenuated TMEM16J‐function upsets regulation and probably compartmentalization of intracellular Ca^2+^. Expression of TMEM16J affects transcription of cytokines, possibly by affecting cytosolic Ca^2+^. Although intracellular Ca^2+^ affects cytokine gene expression by controlling major transcriptional regulators such as NFAT, MEF2, and DREAM,^42^ this does not rule out the calcium‐independent effects of TMEM16A and TMEM16J‐T604A, respectively. Moreover, the pronounced impact of TMEM16J on both intracellular Ca^2+^ and expression of cytokines suggests a direct link but does not exclude additional pathways. Compromised organ function is also seen for the paralogue TMEM16D.^20^, ^34^, ^43^ In the zona glomerulosa of adrenal glands, TMEM16D is one of the most common proteins and limits aldosterone secretion and cell proliferation.^43^ Organ disease that occurs through variant expression of SIGIRR or TMEM16J leads to hyperinflammatory responses by tissue malfunction and/or abnormal T‐cell response.^4^ Interestingly, decreased numbers of SIGIRR‐positive CD4+ T‐helper cells are found in patients with systemic lupus erythematosus.^44^ Intracellular Ca^2+^ is central to T‐cell response and formation of the immunological synapse.^45^, ^46^ It is, therefore, interesting to note, that TMEM16J is expressed at particularly high levels in T‐helper cells and other specialized T‐cells (https://www.proteinatlas.org/ENSG00000185101‐ANO9/immune+cell). Modulation of TMEM16J‐activity by small molecules may, therefore, provide a novel toolbox to control T‐cell response and inflammation.

## AUTHOR CONTRIBUTIONS

Conceptualization: Rainer Schreiber, Khaoula Talbi, Jiraporn Ousingsawat, and Karl Kunzelmann; methodology: Rainer Schreiber, Khaoula Talbi, and Jiraporn Ousingsawat; validation: Rainer Schreiber, Khaoula Talbi, Jiraporn Ousingsawat, and Karl Kunzelmann; formal analysis: Rainer Schreiber, Khaoula Talbi, Jiraporn Ousingsawat, and Karl Kunzelmann; investigation: Rainer Schreiber, Khaoula Talbi, Jiraporn Ousingsawat, and Karl Kunzelmann; data curation: Rainer Schreiber, Khaoula Talbi, Jiraporn Ousingsawat, and Karl Kunzelmann; writing: Rainer Schreiber, Khaoula Talbi, Jiraporn Ousingsawat, and Karl Kunzelmann.

## DISCLOSURES

All the authors declared no competing interests.

## Supporting information


Figure S1

## Data Availability

Data that support the findings of this study are available from the corresponding author upon reasonable request.

## References

[1] Lang T , Mansell A . The negative regulation of toll‐like receptor and associated pathways. Immunol Cell Biol. 2007;85:425‐434. 17621314 10.1038/sj.icb.7100094

[2] Wald D , Qin J , Zhao Z , et al. SIGIRR, a negative regulator of toll‐like receptor‐interleukin 1 receptor signaling. Nat Immunol. 2003;4:920‐927. 12925853 10.1038/ni968

[3] Horne DJ , Randhawa AK , Chau TT , et al. Common polymorphisms in the PKP3‐SIGIRR‐TMEM16J gene region are associated with susceptibility to tuberculosis. J Infect Dis. 2012;205:586‐594. 22223854 10.1093/infdis/jir785 PMC3266131

[4] Garlanda C , Anders HJ , Mantovani A . TIR8/SIGIRR: an IL‐1R/TLR family member with regulatory functions in inflammation and T cell polarization. Trends Immunol. 2009;30:439‐446. 19699681 10.1016/j.it.2009.06.001

[5] Sampath V , Menden H , Helbling D , et al. SIGIRR genetic variants in premature infants with necrotizing enterocolitis. Pediatrics. 2015;135:e1530‐e1534. 25963006 10.1542/peds.2014-3386 PMC4444800

[6] Xiao H , Gulen MF , Qin J , et al. The toll‐interleukin‐1 receptor member SIGIRR regulates colonic epithelial homeostasis, inflammation, and tumorigenesis. Immunity. 2007;26:461‐475. 17398123 10.1016/j.immuni.2007.02.012

[7] Noris M , Cassis P , Azzollini N , et al. The toll‐IL‐1R member Tir8/SIGIRR negatively regulates adaptive immunity against kidney grafts. J Immunol. 2009;183:4249‐4260. 19734209 10.4049/jimmunol.0803549

[8] Pedemonte N , Galietta LJ . Structure and function of TMEM16 proteins (anoctamins). Physiol Rev. 2014;94:419‐459. 24692353 10.1152/physrev.00039.2011

[9] Kim H , Kim H , Lee J , et al. Anoctamin 9/TMEM16J is a cation channel activated by cAMP/PKA signal. Cell Calcium. 2018;71:75‐85. 29604966 10.1016/j.ceca.2017.12.003

[10] Gyobu S , Ishihara K , Suzuki J , Segawa K , Nagata S . Characterization of the scrambling domain of the TMEM16 family. Proc Natl Acad Sci U S A. 2017;114:6274‐6279. 28559311 10.1073/pnas.1703391114 PMC5474828

[11] Kim H , Kim H , Nguyen LT , et al. Amplification of olfactory signals by Anoctamin 9 is important for mammalian olfaction. Prog Neurobiol. 2022;219:102369. 36330924 10.1016/j.pneurobio.2022.102369

[12] Ferenbach D , Hughes J . Macrophages and dendritic cells: what is the difference? Kidney Int. 2008;74:5‐7. 18560360 10.1038/ki.2008.189

[13] Gupta A , Montepiedra G , Gupte A , et al. Low vitamin‐D levels combined with PKP3‐SIGIRR‐TMEM16J host variants is associated with tuberculosis and death in HIV‐infected and ‐exposed infants. PLoS ONE. 2016;11:e0148649. 26872154 10.1371/journal.pone.0148649 PMC4752266

[14] Stanzick KJ , Li Y , Schlosser P , et al. Discovery and prioritization of variants and genes for kidney function in >1.2 million individuals. Nat Commun. 2021;12:4350. 34272381 10.1038/s41467-021-24491-0 PMC8285412

[15] Eckardt KU , Coresh J , Devuyst O , et al. Evolving importance of kidney disease: from subspecialty to global health burden. Lancet. 2013;382:158‐169. 23727165 10.1016/S0140-6736(13)60439-0

[16] Lech M , Avila‐Ferrufino A , Allam R , et al. Resident dendritic cells prevent postischemic acute renal failure by help of single Ig IL‐1 receptor‐related protein. J Immunol. 2009;183:4109‐4118. 19692646 10.4049/jimmunol.0900118

[17] Molgora M , Bonavita E , Ponzetta A , et al. IL‐1R8 is a checkpoint in NK cells regulating anti‐tumour and anti‐viral activity. Nature. 2017;551:110‐114. 29072292 10.1038/nature24293 PMC5768243

[18] Liu G , Honisch S , Liu G , et al. Up‐regulation of Orai1 expression and store operated Ca(2+) entry following activation of membrane androgen receptors in MCF‐7 breast tumor cells. BMC Cancer. 2015;15:995. 26690689 10.1186/s12885-015-2014-2 PMC4687293

[19] Smith KD . Toll‐like receptors in kidney disease. Curr Opin Nephrol Hypertens. 2009;18:189‐196. 19352178 10.1097/MNH.0b013e32832a1d5f PMC2896868

[20] Cabrita I , Benedetto R , Fonseca A , et al. Differential effects of anoctamins on intracellular calcium signals. FASEB J. 2017;31:2123‐2134. 28183802 10.1096/fj.201600797RR

[21] Cabrita I , Buchholz B , Schreiber R , Kunzelmann K . TMEM16A drives renal cyst growth by augmenting Ca(2+) signaling in M1 cells. J Mol Med (Berl). 2020;98:659‐671. 32185407 10.1007/s00109-020-01894-y PMC7220898

[22] Grynkiewicz G , Poenie M , Tsien RY . A new generation of Ca^2+^ indicators with greatly improved fluorescence properties. J Biol Chem. 1985;260:3440‐3450. 3838314

[23] Wu J , Prole DL , Shen Y , et al. Red fluorescent genetically encoded Ca^2+^ indicators for use in mitochondria and endoplasmic reticulum. Biochem J. 2014;464:13‐22. 25164254 10.1042/BJ20140931 PMC4214425

[24] Chen NK , Chong TW , Loh HL , et al. Negative regulatory responses to metabolically triggered inflammation impair renal epithelial immunity in diabetes mellitus. J Mol Med (Berl). 2013;91:587‐598. 23149823 10.1007/s00109-012-0969-x PMC3644409

[25] Kruger WA , Monteith GR , Poronnik P . The plasma membrane Ca(2+)‐ATPase: regulation by PSD‐95/Dlg/Zo‐1 scaffolds. Int J Biochem Cell Biol. 2010;42:805‐808. 20100590 10.1016/j.biocel.2010.01.023

[26] Schreiber R , Ousingsawat J , Kunzelmann K . Targeting of intracellular TMEM16 proteins to the plasma membrane and activation by purinergic signaling. Int J Mol Sci. 2020;21:4065. 32517157 10.3390/ijms21114065 PMC7312528

[27] Tian Y , Schreiber R , Kunzelmann K . Anoctamins are a family of Ca^2+^ activated Cl^−^ channels. J Cell Sci. 2012;125:4991‐4998. 22946059 10.1242/jcs.109553

[28] Reichhart N , Schoberl S , Keckeis S , et al. Anoctamin‐4 is a bona fide Ca(2+)‐dependent non‐selective cation channel. Sci Rep. 2019;9:2257. 30783137 10.1038/s41598-018-37287-y PMC6381168

[29] Warth R , Hamm K , Bleich M , et al. Molecular and functional characterization of the small Ca^2+^‐regulated K^+^ channel (rSK4) of colonic crypts. Pflügers Arch. 1999;438:437‐444. 10519135 10.1007/s004249900059

[30] Feske S , Wulff H , Skolnik EY . Ion channels in innate and adaptive immunity. Annu Rev Immunol. 2015;33:291‐353. 25861976 10.1146/annurev-immunol-032414-112212 PMC4822408

[31] Giannoudaki E , Stefanska AM , Lawler H , et al. SIGIRR negatively regulates IL‐36‐driven Psoriasiform inflammation and neutrophil infiltration in the skin. J Immunol. 2021;207:651‐660. 34253575 10.4049/jimmunol.2100237

[32] Molgora M , Supino D , Mantovani A , Garlanda C . Tuning inflammation and immunity by the negative regulators IL‐1R2 and IL‐1R8. Immunol Rev. 2018;281:233‐247. 29247989 10.1111/imr.12609 PMC5922415

[33] Ji Q , Shi S , Guo S , et al. Activation of TMEM16A by natural product canthaxanthin promotes gastrointestinal contraction. FASEB J. 2020;34:13430‐13444. 32812278 10.1096/fj.202000443RR

[34] Jha A , Chung WY , Vachel L , et al. Anoctamin 8 tethers endoplasmic reticulum and plasma membrane for assembly of Ca(2+) signaling complexes at the ER/PM compartment. EMBO J. 2020;38:e101452. 10.15252/embj.2018101452 PMC6576175 31061173

[35] Lemos FO , Bultynck G , Parys JB . A comprehensive overview of the complex world of the endo‐ and sarcoplasmic reticulum Ca(2+)‐leak channels. Biochim Biophys Acta Mol Cell Res. 2021;1868:119020. 33798602 10.1016/j.bbamcr.2021.119020

[36] Kunzelmann K , Cabrita I , Wanitchakool P , et al. Modulating Ca^2+^ signals: a common theme for TMEM16, Ist2, and TMC. Pflügers Arch. 2016;468:475‐490. 26700940 10.1007/s00424-015-1767-4

[37] Kralt A , Carretta M , Mari M , et al. Intrinsically disordered linker and plasma membrane‐binding motif sort Ist2 and Ssy1 to junctions. Traffic. 2015;16:135‐147. 25409870 10.1111/tra.12243

[38] Barreda D , Gutiérrez‐González LH , Martínez‐Cordero E , Cabello‐Gutiérrez C , Chacón‐Salinas R , Santos‐Mendoza T . The scribble complex PDZ proteins in immune cell polarities. J Immunol Res. 2020;2020:5649790. 32411799 10.1155/2020/5649790 PMC7210543

[39] Marziali F , Dizanzo MP , Cavatorta AL , Gardiol D . Differential expression of DLG1 as a common trait in different human diseases: an encouraging issue in molecular pathology. Biol Chem. 2019;400:699‐710. 30517074 10.1515/hsz-2018-0350

[40] Kruger WA , Yun CC , Monteith GR , Poronnik P . Muscarinic‐induced recruitment of plasma membrane Ca^2+^‐ATPase involves PSD‐95/Dlg/Zo‐1‐mediated interactions. J Biol Chem. 2009;284:1820‐1830. 19017653 10.1074/jbc.M804590200 PMC2615496

[41] Luck K , Kim DK , Lambourne L , et al. A reference map of the human binary protein interactome. Nature. 2020;580:402‐408. 32296183 10.1038/s41586-020-2188-x PMC7169983

[42] Savignac M , Mellström B , Naranjo JR . Calcium‐dependent transcription of cytokine genes in T lymphocytes. Pflugers Arch. 2007;454:523‐533. 17334777 10.1007/s00424-007-0238-y

[43] Maniero C , Scudieri P , Haris Shaikh L , et al. ANO4 (Anoctamin 4) is a novel marker of zona glomerulosa that regulates stimulated aldosterone secretion. Hypertension. 2019;74:1152‐1159. 31564164 10.1161/HYPERTENSIONAHA.119.13287 PMC6791498

[44] Wang DY , Su C , Chen GM , et al. The decreased frequency of SIGIRR‐positive CD4+ T cells in peripheral blood of patients with SLE and its correlation with disease activity. Mol Biol Rep. 2015;42:423‐430. 25287661 10.1007/s11033-014-3783-4

[45] Trebak M , Kinet JP . Calcium signalling in T cells. Nat Rev Immunol. 2019;19:154‐169. 30622345 10.1038/s41577-018-0110-7 PMC6788797

[46] Babich A , Burkhardt JK . Coordinate control of cytoskeletal remodeling and calcium mobilization during T‐cell activation. Immunol Rev. 2013;256:80‐94. 24117814 10.1111/imr.12123 PMC3824381

[47] Cha HJ , Jung MS , Ahn do W , et al. Silencing of MUC8 by siRNA increases P2Y₂‐induced airway inflammation. Am J Physiol Lung Cell Mol Physiol. 2015;308:L495‐L502. 25575516 10.1152/ajplung.00332.2014

[48] Vanderstocken G , Van de Paar E , Robaye B , et al. Protective role of P2Y2 receptor against lung infection induced by pneumonia virus of mice. PloS One. 2012;7:e50385. 23185614 10.1371/journal.pone.0050385 PMC3503929

