# Supplementary material for: A TMEM16J variant leads to dysregulated cytosolic calcium which may lead to renal disease
Source: FASEB J. 2022 Dec 15;37(1):e22683. doi: 10.1096/fj.202200968R (PMC13281847; doi:10.1096/fj.202200968R)
Supplement: Supplementary file 1 — Figure S1 [file FSB2-37-e22683-s001.pdf]

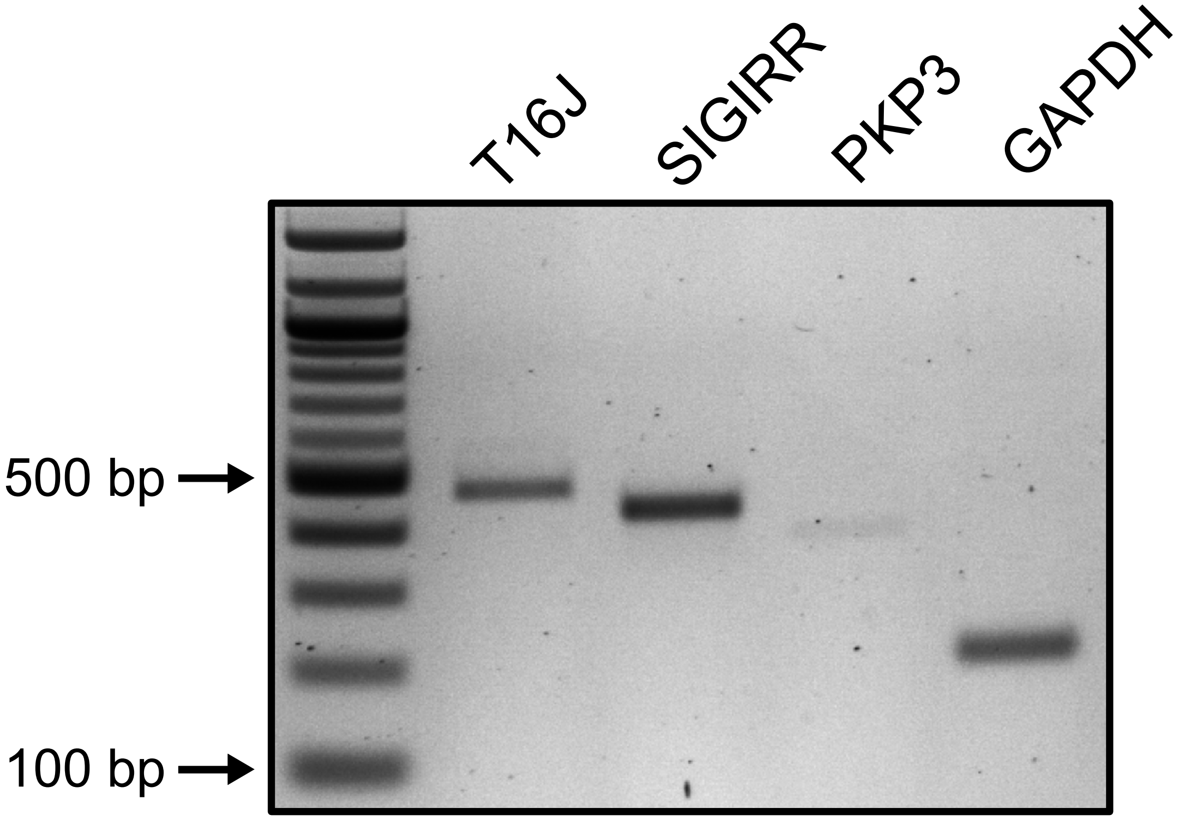


**Supplementary Figure 1.** *Expression of TMEM16J, SIGIRR and PKP3 in human kidney.* Expression of TMEM16J (T16J), single immunoglobulin interleukin-1 related receptor (SIGIRR), and plakophilin 3 (PKP3) in human kidney, as detected by RT-PCR.

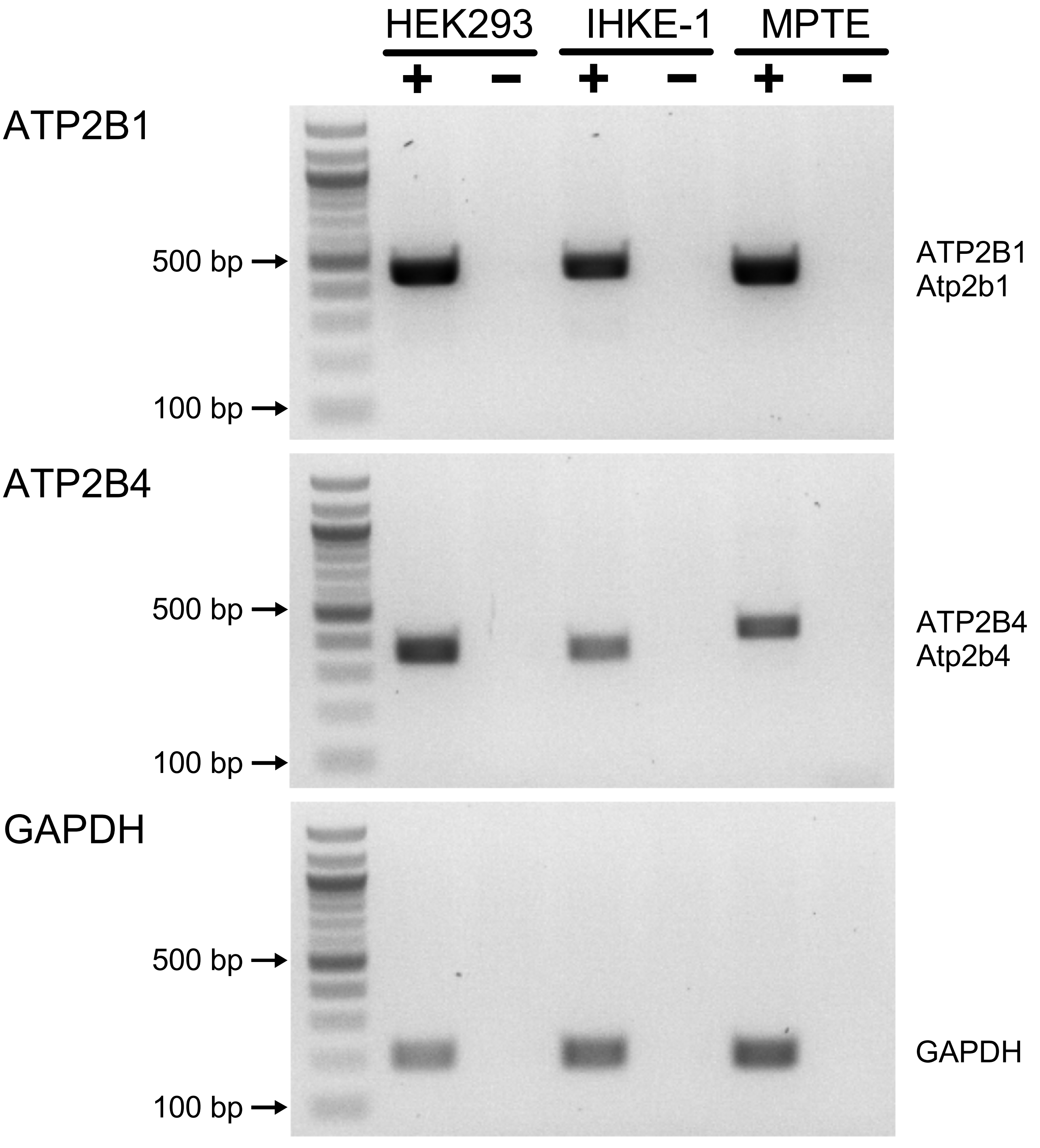


**Supplementary Figure 2.** *Expression of PMCAs.* Expression of plasma membrane $Ca^{2+}$-ATPases (PMCA; ATP2B1, ATP2B2) in HEK293 cells, immortalized human kidney epithelial (IHKE-1) cells, and primary proximal tubular epithelial (MPTE) cells, as detected by RT-PCR.

**a**

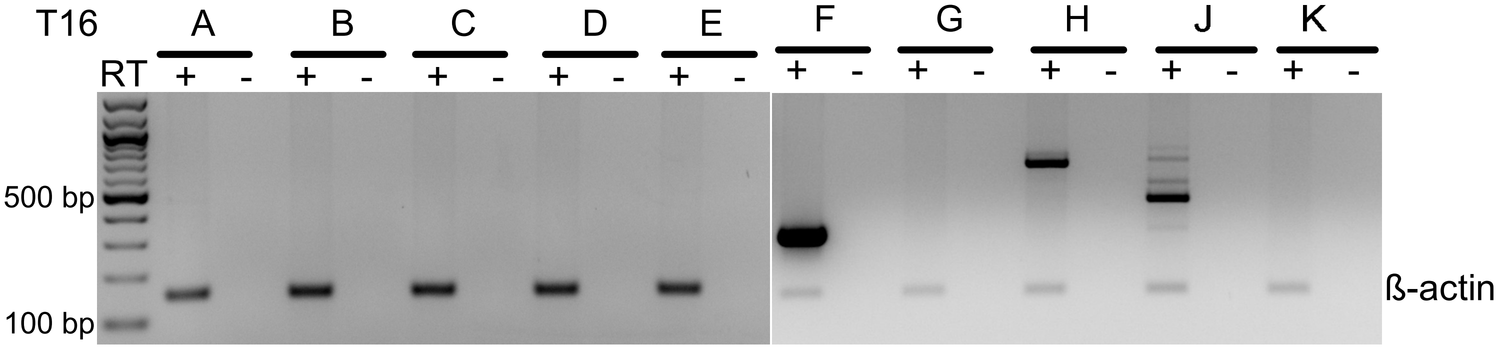


**b**

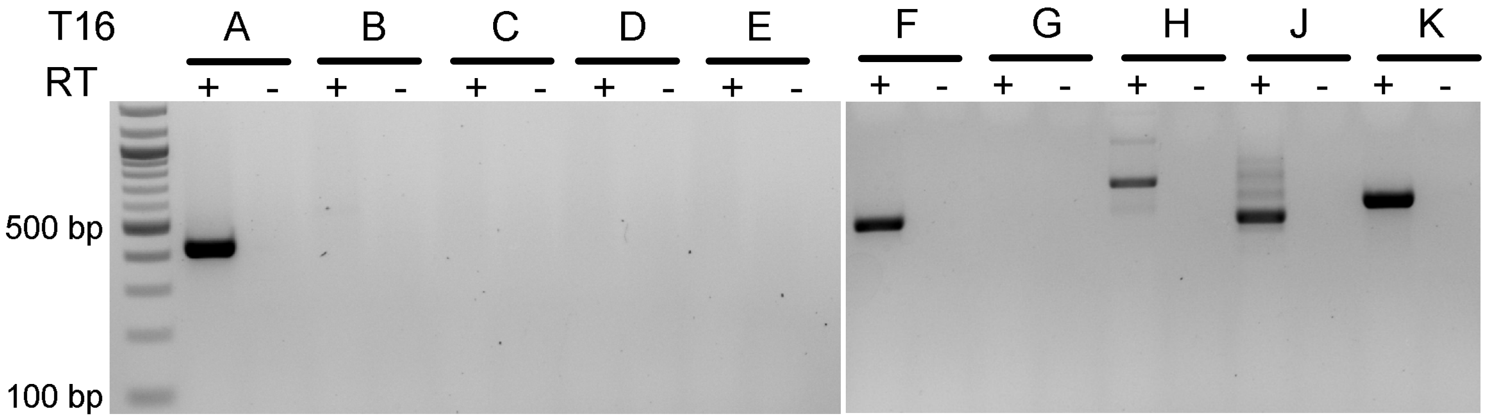


**Supplementary Figure 3.** *Expression of TMEM16 proteins in IHKE-1 and MPTE cells.* Expression of TMEM16 (T16) A-K in **a**) immortalized human kidney epithelial (IHKE-1) and **b**) mouse primary proximal tubular epithelial (MPTE) cells as detected by RT-PCR.

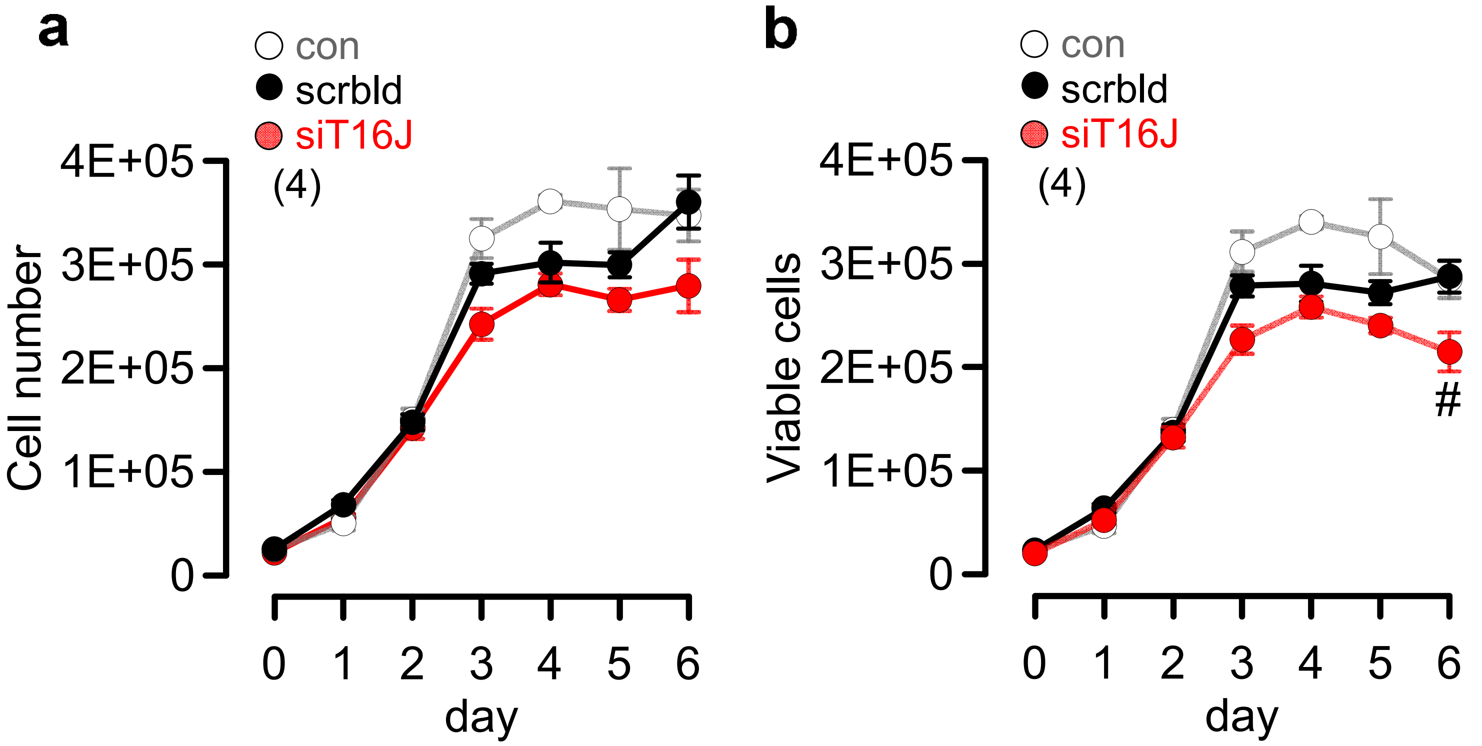


**Supplementary Figure 4**. *Cell proliferation and cell death is not affected by expression of TMEM16J in IHKE-1 cells.* **a**) Cell proliferation assed by cell counting of non-transfected (con) cells, and cells treated with scrambled RNA (scrbld) or siRNA for TMEM16J (siT16J). **b**) Number of viable cells. Mean ± SEM (number of experiments).

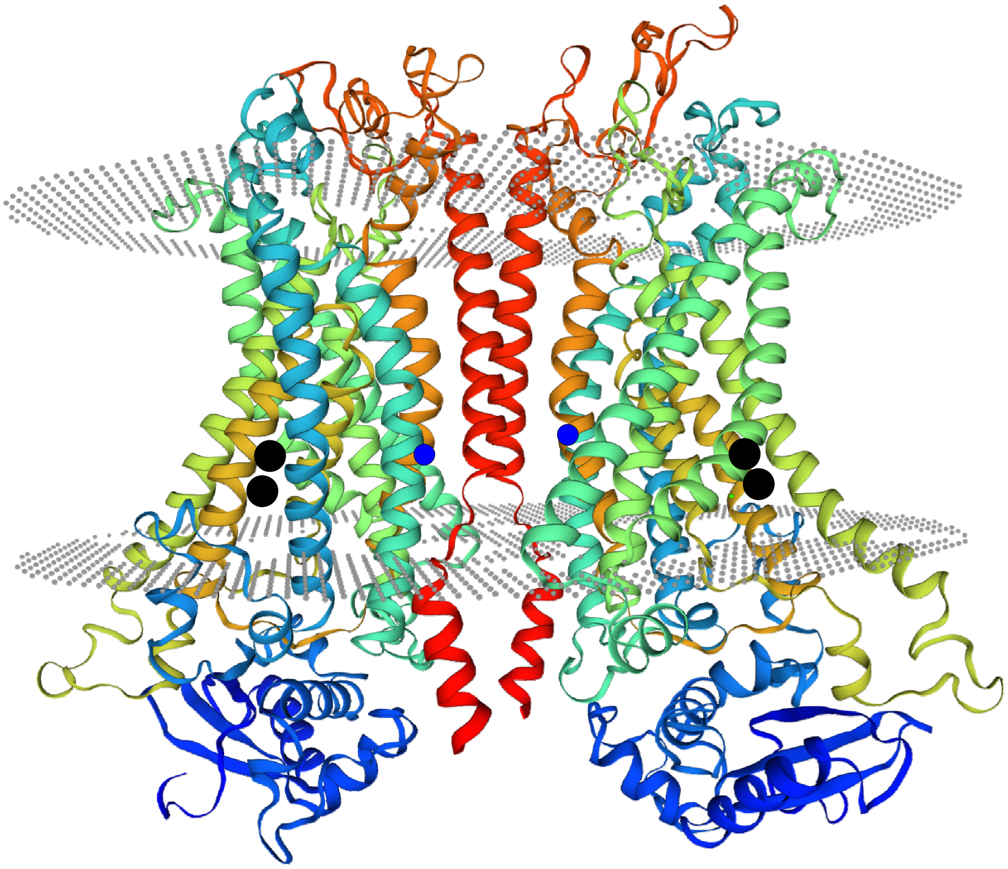

**Supplementary Figure 5**. *Putative structure of TMEM16J.* Putative structure of TMEM16J based on mouseTMEM16F. Sequence identity 35.43% (Waterhouse, A., Bertoni, M., Bienert, S., Studer, G., Tauriello, G., Gumienny, R., Heer, F.T., de Beer, T.A.P., Rempfer, C., Bordoli, L., Lepore, R., Schwede, T. SWISS-MODEL: homology modelling of protein structures and complexes. Nucleic Acids Res. 46(W1), W296-W303 (2018). Black filled circles: Approximate location of $Ca^{2+}$ binding sites in each subunit. Blue filled circles: Approximate location of T604 in TMD9 (orange helix).

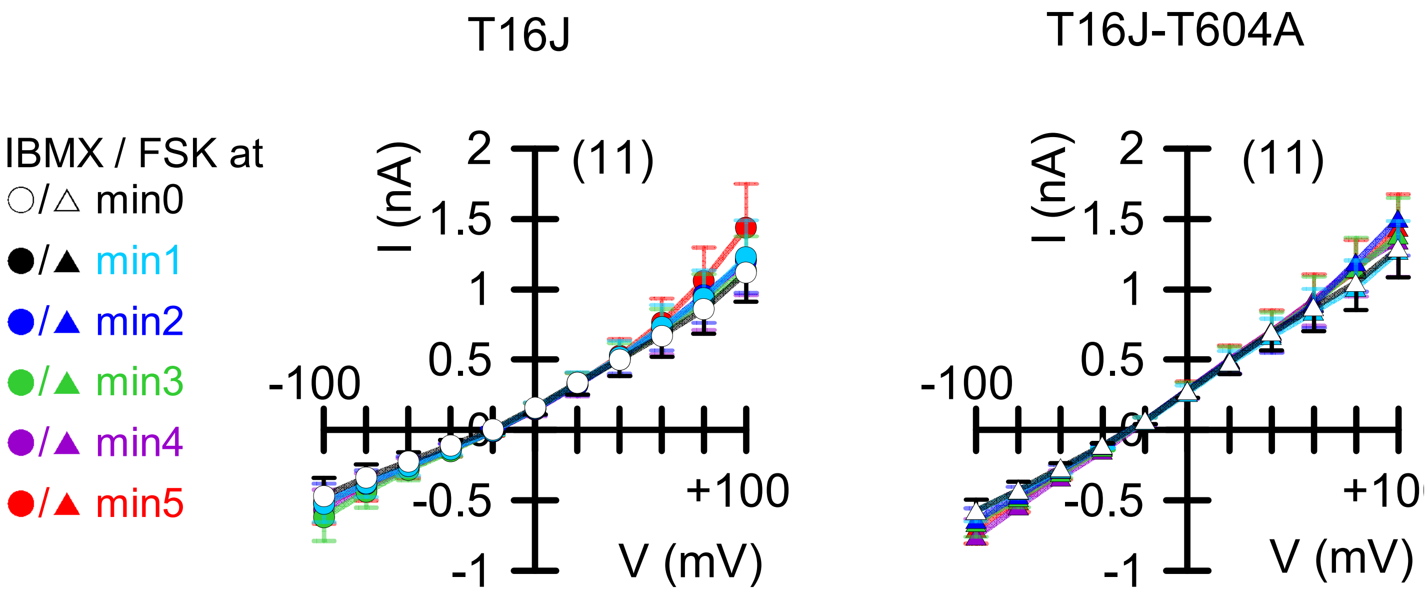


**Supplementary Figure 6.** *No activation of TMEM16J by increase in intracellular cAMP.* TMEM16J (T16J) and the variant TMEM16J-T604A (T16J-T604A) were expressed in HEK293 cells. Over a time period of 5 min intracellular cAMP was enhanced by stimulation with 3-Isobutyl-1-methylxanthine (IBMX; 100 µM) and forskolin (2 µM), which did not activate additional whole cell currents. Current/voltage relationships with mean ± SEM (number of experiments).

**a**

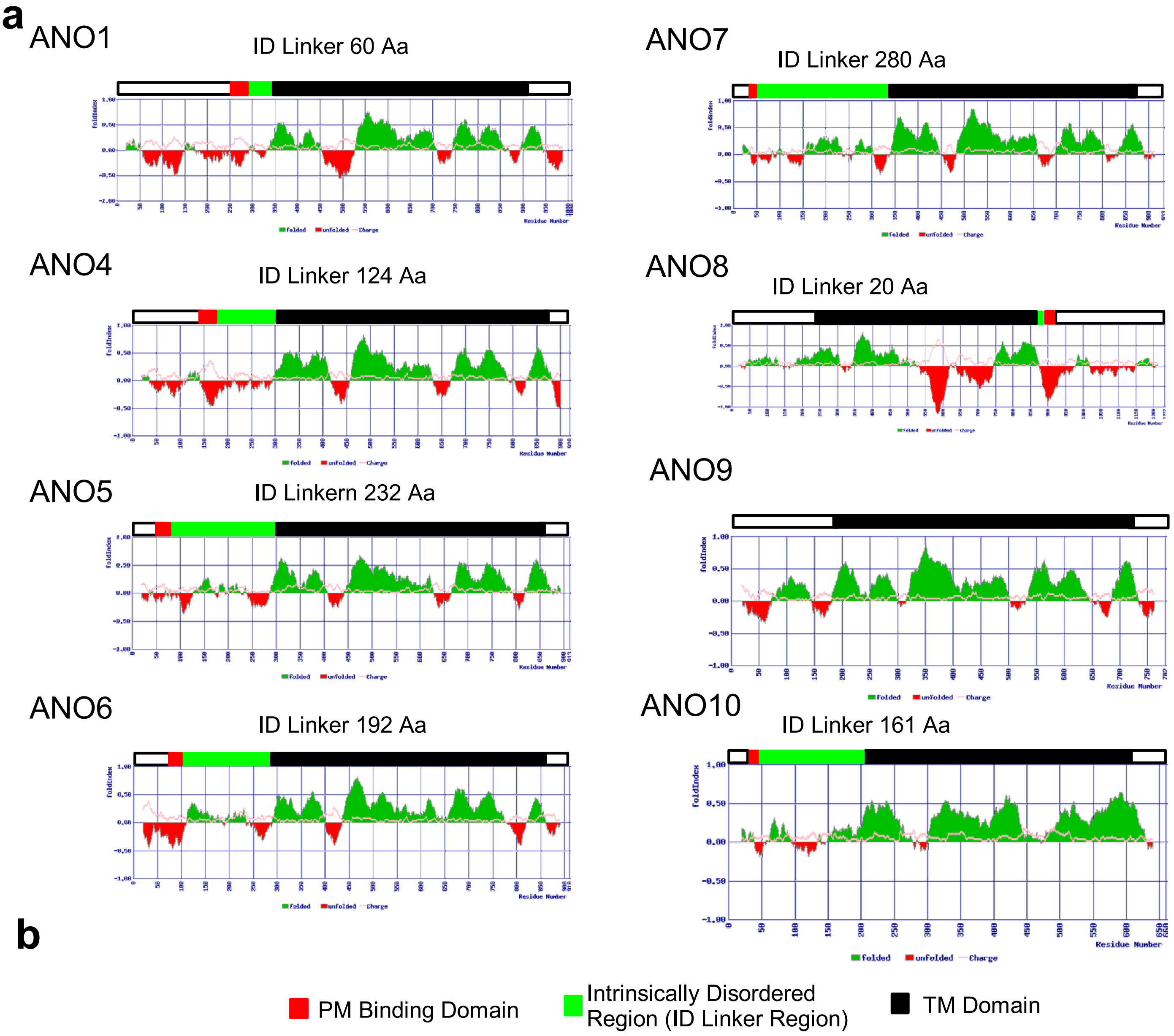


**b**

Human ANO9

MQGEESLRILVEPEGDSFPLMEISTCETEASEQWDYVLVAQRHTQRDPRQARQQQFLEELRRKGFHIKVI
RDQKQVFFGIRADNSVFGLYRTLLLEPEGPAPHAELAAPTTIPVTTSLRIRIVNFVVMNNKTSAGETFED
LMKDGVFEARFPLHKGEGRLKKTWARWRHMFREQPVDEIRNYFGEKVALYFVWLGWYTYMLVPAALTGLL
VFLSGFSLFEASQISKEICEAHDILMCPLGDHSRRYQRLSETCTFAKLTHLFDNDGTVVFAIFMALWATV
FLEIWKRQRARVVLHWDLYVWDEEQEEMALQLINCPDYKLRPYQHSYLRSTVILVLTLLMICLMIGMAHV
LVVYRVLASALFSSSAVPFLEEQVTTAVVVTGALVHYVTIIIMTKINRCVALKLCDFEMPRTFSERESRF
TIRFFTLQFFTHFSSLIYIAFILGRINGHPGKSTRLAGLWKLEECHASGCMMDLFVQMAIIMGLKQTLSN
CVEYLVPWVTHKCRSLRASESGHLPRDPELRDWRRNYLLNPVNTFSLFDEFMEMMIQYGFTTIFVAAFPL
APLLALFSNLVEIRLDAIKMVWLQRRLVPRKAKDIGTWLQVLETIGVLAVIANGMVIAFTSEFIPRVVYK
YRYSPCLKEGNSTVDCLKGYVNHSLSVFHTKDFQDPDGIEGSENVTLCRYRDYRNPPDYNFSEQFWFLLA
IRLAFVILFEHVALCIKLIAAWFVPDIPQSVKNKVLEVKYQRLREKMWHGRQRLGGVGAGSRPPMPAHPT
PASIFSARSTDV

**Supplementary Figure 7:** *TMEM16J does not contain a cluster of positively charged amino acids in either N- or C terminus.* **a**) Comparison of potential plasma membrane (PM) binding domains, intrinsically disordered regions and transmembrane (TM) domain in different TMEM16 (anoctamin; ANO) paralogs. **b**) Amino acid sequence of human TMEM16J (ANO9).

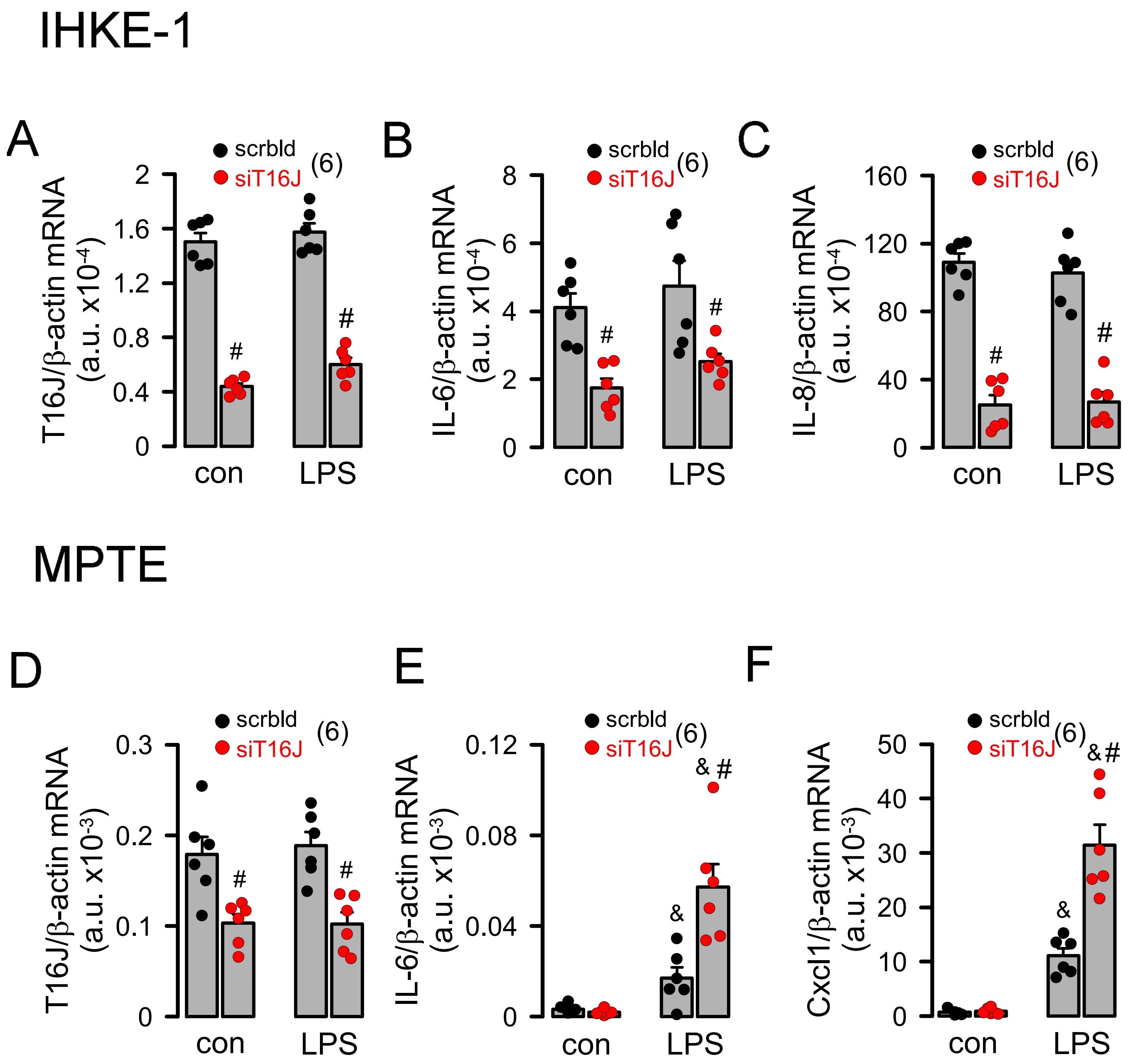


**Supplementary Figure 8.** *Real-time RT-PCR of TMEM16J and interleukins in IHKE-1 and MPTE cells.* A) Real-time PCR indicating suppression of TMEM16J-T604A expression by siRNA in IHKE-1 cells under control conditions and in the presence of LPS. B,C) Effect of TMEM16J-T604A knockdown (si-T16J) on expression of endogenous IL-6 and IL-8 in IHKE-1 cells. D) Real-time PCR indicating suppression of TMEM16J-T604A expression by siRNA in MPTE cells, under control conditions and in the presence of LPS. E,F) Effect of TMEM16J knockdown (siT16J) on expression of endogenous IL-6 and IL-8 in MPTE cells.

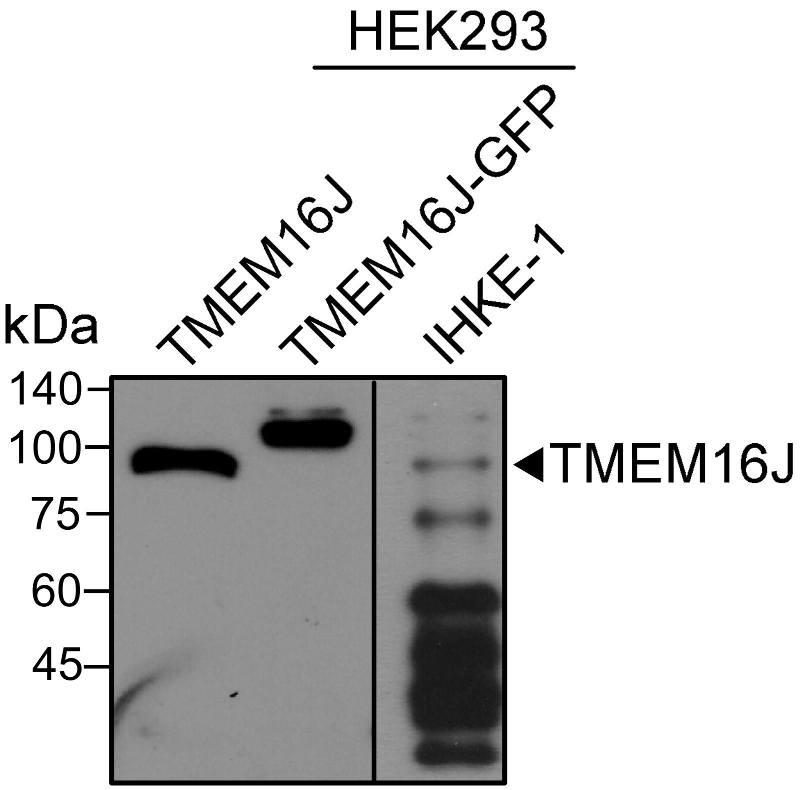


**Supplementary Figure 9.** *Western blotting of TMEM16J.* Analysis of TMEM16J overexpressed in HEK293 cells and TMEM16J expressed endogenously in IHKE-1 cells.

Fig. 5a

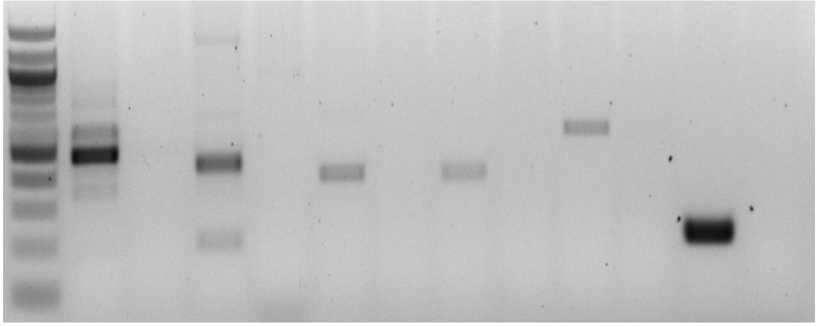

Fig. S1

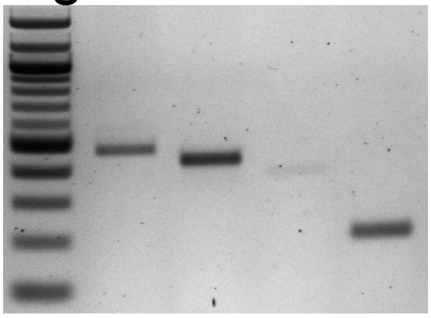

Fig. 6a

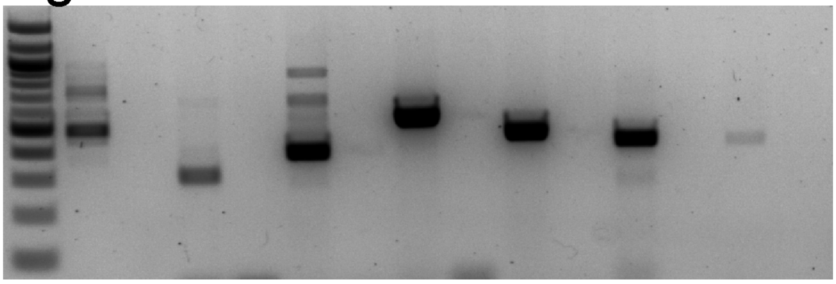

Fig. S3

a

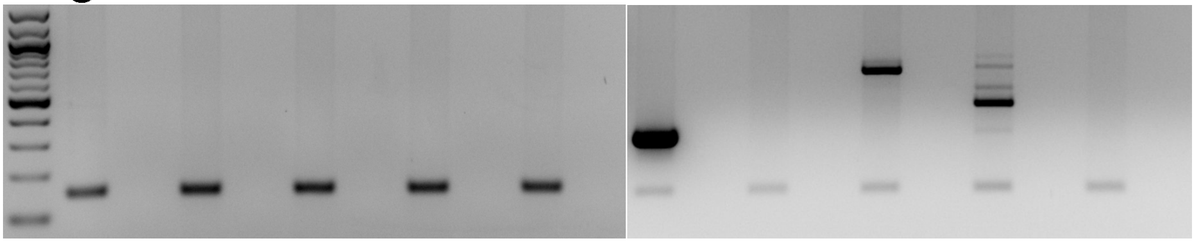

b

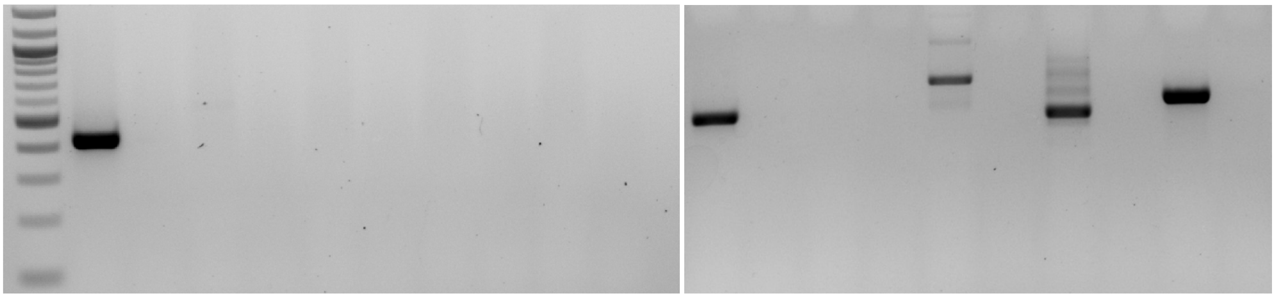

Fig. S2

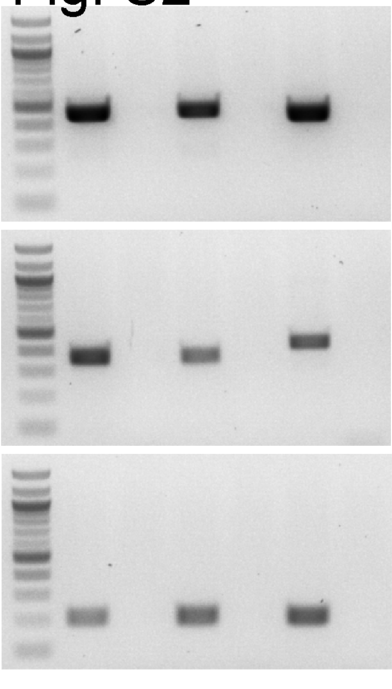

Fig. S9

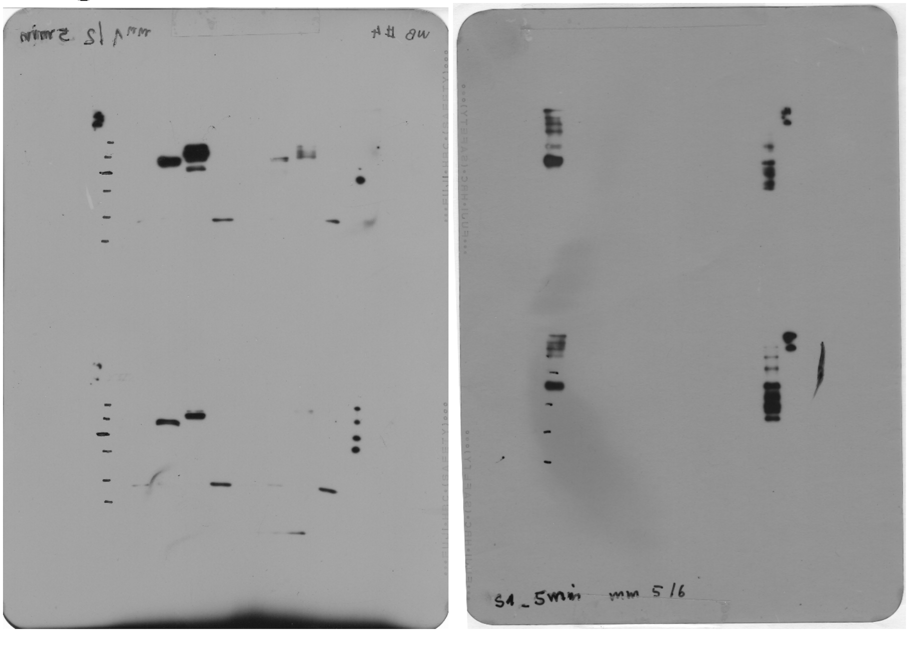


**Supplementary Figure 10:** *Uncropped blots*.
